# Seasonality of bovine respiratory disease and mitigation techniques used in receiving beef cattle in the last 10 years: a meta-analysis

**DOI:** 10.1093/tas/txag152

**Published:** 2026-10-05

**Authors:** Robin A Cheek, E B Kegley, S M Howe, A Goswami, J G Powell

**Affiliations:** Department of Animal Science, University of Arkansas System Division of Agriculture, Fayetteville, AR 72701, United States; Department of Animal Science, University of Arkansas System Division of Agriculture, Fayetteville, AR 72701, United States; Department of Animal Science, University of Arkansas System Division of Agriculture, Fayetteville, AR 72701, United States; Department of Animal Science, University of Arkansas System Division of Agriculture, Fayetteville, AR 72701, United States; Department of Animal Science, University of Arkansas System Division of Agriculture, Fayetteville, AR 72701, United States

**Keywords:** beef cattle, receiving, morbidity, meta-analysis

## Abstract

Despite recent advances in technology and biological, nutritional, and pharmaceutical products designed to mitigate disease, bovine respiratory disease (BRD) remains the leading cause of feedlot morbidity. The objectives were to (1) quantify the effects of BRD interventions that have been reported in the literature within the United States for the previous decade, and (2) to investigate seasonal effects on BRD incidence. A detailed literature search was conducted using Preferred Reporting Items for Systematic Reviews and Meta-analyses methods. A total of 103 records (56 studies) met criteria by providing percentages of cattle treated at least once for BRD and reporting a control. Available data were extracted from each study for control groups and treatment groups and a logs risk ratio of both metrics (ai = controlBRD, bi = controlHealthy, ci = treatmentBRD, di = treatmentHealthy) was calculated for each record using the escalc function in the R package metafor (v4.8) in R (v4.5). A multi-level meta-analytical (MLMA) model was used to determine overall effects on BRD morbidity because data non-independence existed. The MLMA model yielded a pooled estimate for log ratios of 0.07 (*P* = 0.09) and indicated the presence of heterogeneity (Q = 336, *P* < 0.0001), suggesting that BRD incidence tended to decrease in treated cattle. When testing study-level characteristics, season (*P* = 0.02) and study location (*P* = 0.02) influenced overall BRD incidence. Metaphylactic programs (ln*OR = *0.17; *P* = 0.05) and technological diagnostic tools (ln*OR = *0.40; *P* = 0.06) modestly decreased overall BRD incidence. Metaphylactic experiments evaluating antimicrobial resistance reduced BRD incidence (ln*OR = *1.28; *P* = 0.01), specifically studies including tulathromycin. Nutritional experiments testing additional vitamin E (ln*OR = *−0.32; *P* = 0.05) and pelleting feed (ln*OR = *0.34; *P* = 0.08) had the greatest influence on BRD. The Egger’s regression test for funnel plot asymmetry was performed which revealed significant asymmetry (*P* < 0.0001) suggesting evidence of publication bias. To conclude, BRD tended to decrease in cattle that were applied with treatments, but there was great variation among studies, therefore, the magnitude varied. The results support prioritizing management decisions based on environmental and regional conditions. Interventions for BRD in this analysis suggest that metaphylactic programs and technological diagnostics may be more effective in certain contexts.

## Teaser

Incidence of respiratory disease in beef calves tended to decrease in cattle that were applied with mitigation techniques, but there was great variation among studies; therefore, the magnitude of response varied. Results from this meta-analysis support prioritizing environmental factors and suggest that metaphylactic programs and technological diagnostics are, at this time, the most effective respiratory disease interventions.

## Introduction

Within the United States, beef cattle production relies on various segments, which include: seedstock, cow-calf, stocker-feedlot, and packers. Each segment works simultaneously over time to produce a wide variety of products for consumers. Cow-calf operations produce calves that are either sent for pasture (stocker) or forage-based feeding in backgrounding dry lots, which totals up to 60% of weaned calves produced in the United States (Herring 2014; Drouillard 2018). In turn, cow-calf operations produce calves that are transported directly to the feedlot, to be placed on feed for approximately 240 days before the time of slaughter. Most calves are weaned from the dam between 6 and 8 months of age and often sold through local sale barns.

Calves that are placed in North American auction markets are then left at a greater risk of developing respiratory issues due to the predisposing factors like transportation and comingling (Wilson et al. 2017). Bovine respiratory disease (BRD) is a multifactorial syndrome that involves multiple viral and bacterial pathogens. The multifactorial complexity of this disease, coupled with the various interactions of animals, pathogens, and their environment in its pathogenesis, poses significant challenges in effecting disease control (Murray et al. 2016). The prevalence of this disease underlines a fundamental gap in comprehending the host immune response to respiratory infections.

Season has been directly associated with respiratory disease incidence and is likely a proxy for weather conditions, which vary by geographical location. It was concluded in a review studying the pathogenesis of BRD that although the basic features are relatively well understood, the multiple factors involved create combinations and complex interactions between the environment, infectious agents, and the host, which will continue to provide challenges to our understanding and management of BRD (Mosier 2015).

Despite recent advances in technology, biological, nutritional, and pharmaceutical products designed to mitigate disease, BRD remains the leading cause of morbidity, mortality, and economic loss to the cattle industry. Costs associated with mortality, reduction of feed efficiency, and BRD treatment are estimated to cost the industry $800 to $900 million per year in the United States (Centeno-Martinez et al. 2022). Thus, the objectives of this study were to conduct a detailed literature search to (1) examine various mitigation techniques that have been reported within the United States for the previous decade, and (2) to investigate seasonality effects on BRD incidence and severity.

## Materials and methods

### Literature search and data extraction

A detailed literature search was conducted using Preferred Reporting Items for Systematic Reviews and Meta-Analyses *(PRISMA)* methods on 22 October 2025 (Fig. 1; Moher et al. 2009). The search terms “bovine respiratory disease” OR “BRD” were used to search the Mendeley Reference Manager database. This database was searched to acquire literature across various scientific journals. A total of 1327 records were pooled, and 63 duplicates were removed. Four hundred and twenty records were marked as ineligible by automation tools, which included publications before 2015. In turn, there were a total of 483 records that were excluded before screening. Then, 844 records remained eligible to be screened for relevance. A total of 788 records were excluded as they fell outside of the designated selection criteria (outside of the United States, did not report morbidity percentages, or absence of a control group). The remaining records (*n* = 56) met the criteria and were included in the meta-analysis. It is important to note that each study included was reporting naturally occurring incidence of BRD within the receiving period across various study settings (controlled pen trials, experiment station studies, or commercial field studies).

**Figure 1:**
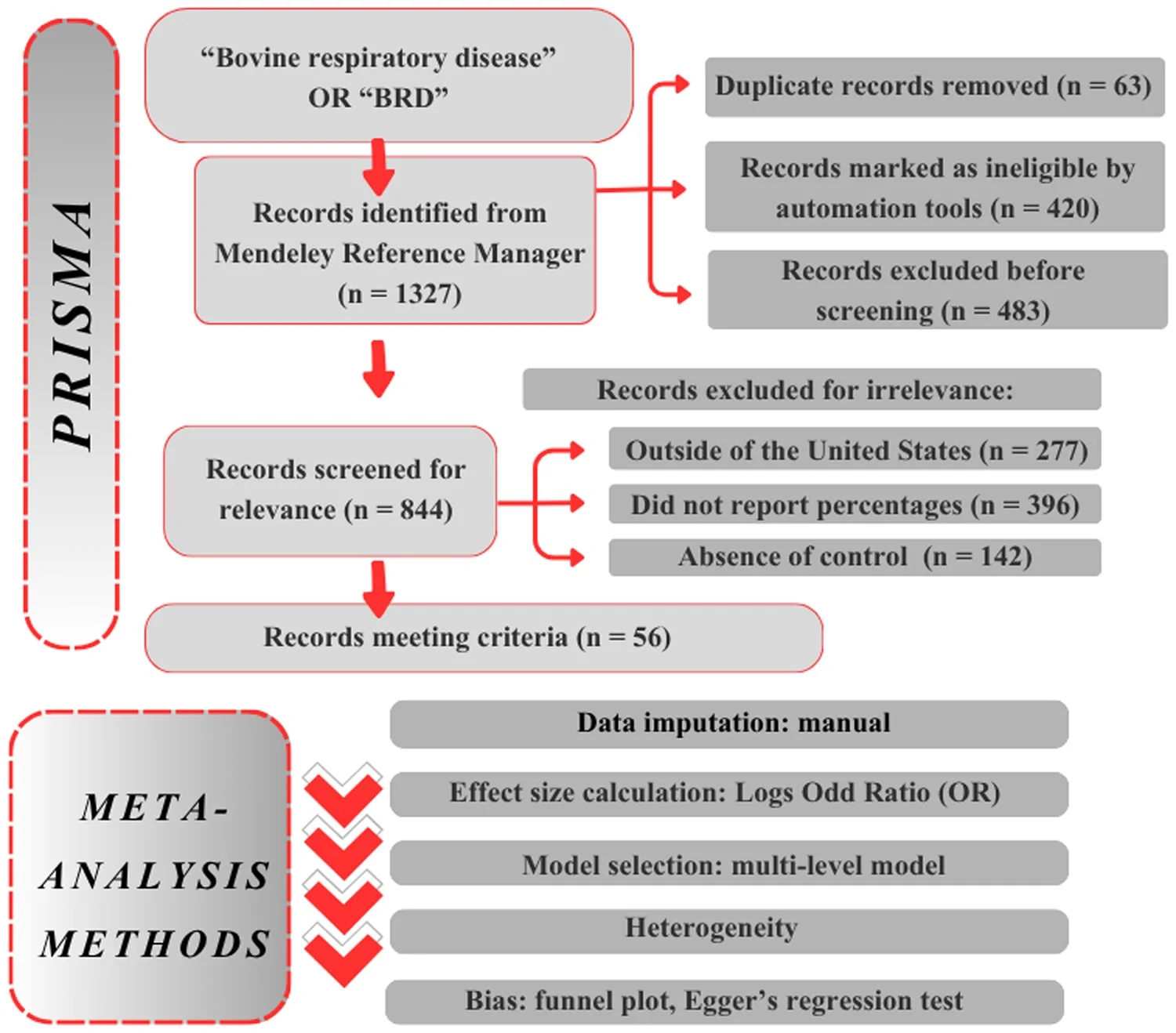
Preferred Reporting Items for Systematic Reviews and Meta-Analyses (PRISMA) methods used on 22 October 2025 (Moher et al. 2009).

Available data (morbidity percentages, number and type of experimental treatments used, sample size, study location, sex, and season) were extracted from each study for control groups and treatment groups. All morbidity data extracted were reported in tables for each control and treatment group (metaphylactic, nutritional, product administration, technology diagnostics, or source) within each publication. The types of studies (BRD mitigation techniques) were as follows: metaphylactic therapy, nutritional intervention, new-product administration, technology diagnostics, and cattle sources. Metaphylactic studies included experiments that were evaluating metaphylactic use of antimicrobials on subsequent BRD incidence. Nutritional studies included experiments evaluating certain dietary treatments on subsequent BRD incidence. Studies termed as “product” evaluated administration of a product in question (vaccinations, meloxicam, maternal bovine appeasing substance, mineral oil, growth implants, or nonsteroidal anti-inflammatory drugs) on subsequent BRD incidence. Studies termed as “technology diagnostics” evaluated new technology systems (electronic stethoscope [Whisper]), remote early disease identification systems, or infrared thermography] to diagnose BRD versus traditional pull-and-treat protocols. Lastly, studies that were termed “source” were experiments evaluating cattle that were derived from 1, 2, or 3 sources and being comingled on subsequent BRD incidence.

The percentage of cattle that were treated at least once with antibiotics for BRD was the only morbidity data extracted. The percentage of cattle considered healthy (not treated with antibiotics for BRD) within an experimental treatment group was calculated using Microsoft Excel (100—percentage of cattle treated at least once for BRD). In addition, to assess baseline risk for BRD overall morbidity percentages associated with BRD were calculated ([total number of cattle treated for BRD/total number of cattle on trial] × 100) using Microsoft Excel for each study. Each study was then assigned 1 of 2 BRD risk factors: 1) below 15% morbidity (LR = low risk), or 2) greater than or equal to 15% morbidity (HR = high risk). Risk factors were determined based on Rojas et al. (2022) recommendations.

### Effect size calculation

A database containing relevant metadata was constructed to calculate individual study effect sizes. A log risk ratio of both metrics (ai = controlBRD, bi = controlHealthy, ci = treatmentBRD, di = treatmentHealthy) was calculated for each record using the escalc function in the R package metafor (v4.8) in R (v4.5; Viechtbauer 2010; R Core Team 2022).

### Meta-analytical model

A multi-level meta-analytical model (*MLMA*) was used to determine the overall effect of treatments (mitigation techniques) on BRD in beef cattle within the past 10 years. Potential non-independence and heterogeneity were accounted for by calculating multiple effect sizes for specific records, as numerous studies reported metrics of interest for more than 2 experimental treatments compared to the control treatment. Therefore, this was accomplished by utilizing the rma.mv function in the metafor package and by setting “random = ∼1 | pub/study,” in which “pub” being the publication and “study” being the study count entry number. Additionally, the model was fitted using restricted likelihood (*REML*). Following, the model was tested for level effects of season (month cattle were received), experimental treatment, study location, sex, and risk. This was done by adding “∼1 | season,” “∼1 | type,” “∼1 | loc,” “∼1 | sex,” and “∼1 | risk” as random effects in the model.

Furthermore, a subset analysis was performed to quantify the effects of nutritional and metaphylactic interventions evaluated within the dataset. Nutritional and metaphylactic interventions were quantified by adding a column to the dataset providing experimental treatment information. The REML model was then tested for level-specific effects when narrowing the dataset to only nutritional and metaphylactic records.

### Residual heterogeneity

To investigate the source of variation in effect sizes among studies, a subgroup analysis was performed, which specifically looked at location, season, study type, sex, sample size, and risk factor. A test for residual heterogeneity was conducted by adjusting the MLMA by setting “mods = ∼ factor(loc)-1,” for example.

### Publication bias

To investigate the publication bias of beef cattle BRD morbidity percentages, the MLMA model was used to generate funnel plots via the funnel function in the metafor package. Potential small-study effects and funnel-plot asymmetry were assessed using a multilevel Egger-type regression analysis, in which the standard error of each effect size was included as a moderator while retaining the hierarchical structure of effect sizes nested within publication.

For all data, significance was declared when *P* ≤ 0.05 and considered a tendency if 0.05 < *P* ≤ 0.10.

## Results

### Literature results, data entry

The literature search resulted in 56 records, which were published research journal articles. The final database constructed for this meta-analysis consisted of 103 data entries. Information from each entry included location (state), the number of treatments (*n*), morbidity percentages (controlBRD, controlHealthy, treatmentBRD, and treatmentHealthy), sample size (*n*), season (month cattle were received), sex (steers, heifers, bulls, or combination), study type (drug, metaphylactic, nutritional, source, and bovine appeasing substance), and risk factor (LR or HR). A detailed list of raw data and variables extracted from each study can be found in Supplementary Table 1, available as supplementary data at *Translational Animal Science* online. All references for each study used to conduct this meta-analysis can be found in Table S2, available as supplementary data at *Translational Animal Science* online.

The effect sizes calculated were the natural logarithms of the odds ratio (ln*OR*) and relative risk. This resulted in a log risk ratio for each record (vi) and the variance of each log risk ratio (vi), which was used in the meta-analysis for weighting each record.

### Random-effects model outcomes

The REML model constructed using the ln*OR* and relative risks calculated revealed a significant positive overall effect. This test indicated that the BRD interventions applied significantly increased the odds of cattle remaining healthy compared to cattle in control groups (lnOR = 0.1009 ± 0.04; *P* = 0.0058). Converting this to the odds ratio (OR) scale, the treatment improved the odds of healthy cattle by approximately 10.6% for those on treatment compared to controls. The pooled estimate for OR was relatively small at 1.106 (*P* = 0.0058; 95% CI: 1.0300–1.1880). Because this resulted in a small effect size, the mitigation techniques used did improve overall health, but the magnitude of this was modest. Because the OR varied widely across the 103 records, this resulted in a large heterogeneity value of 82.76% (*P* < 0.0001). This suggested that true treatment effects differed, and some studies showed a strong benefit, but others showed little or no benefit.

### Multi-level model outcomes

The MLMA model used publication as a high-level grouping factor, which indicated substantial heterogeneity between studies (σ2 = 0.0677), but there was no detectable heterogeneity among effect sizes within studies (σ2 = 0.0000). Therefore, there was strong evidence of overall heterogeneity (*Q* = 336; *P* < 0.0001), which is shown in funnel plots in Fig. 2. The funnel plot reveals slight symmetry reflecting heterogeneity across individual records and the random effects due to the variability at greater levels. The estimated overall effect for log ratios was 0.0719 ± 0.0426 (*P* = 0.0917, 95% CI: −0.0117–0.1555), corresponding to an OR of 1.07 (95% CI: 0.99–1.17). This indicated that there was an overall tendency for the disease interventions evaluated to influence the BRD incidences reported. Therefore, after accounting for the clustering of multiple effect sizes within publications, the treatment effect had decreased and was not significant.

**Figure 2:**
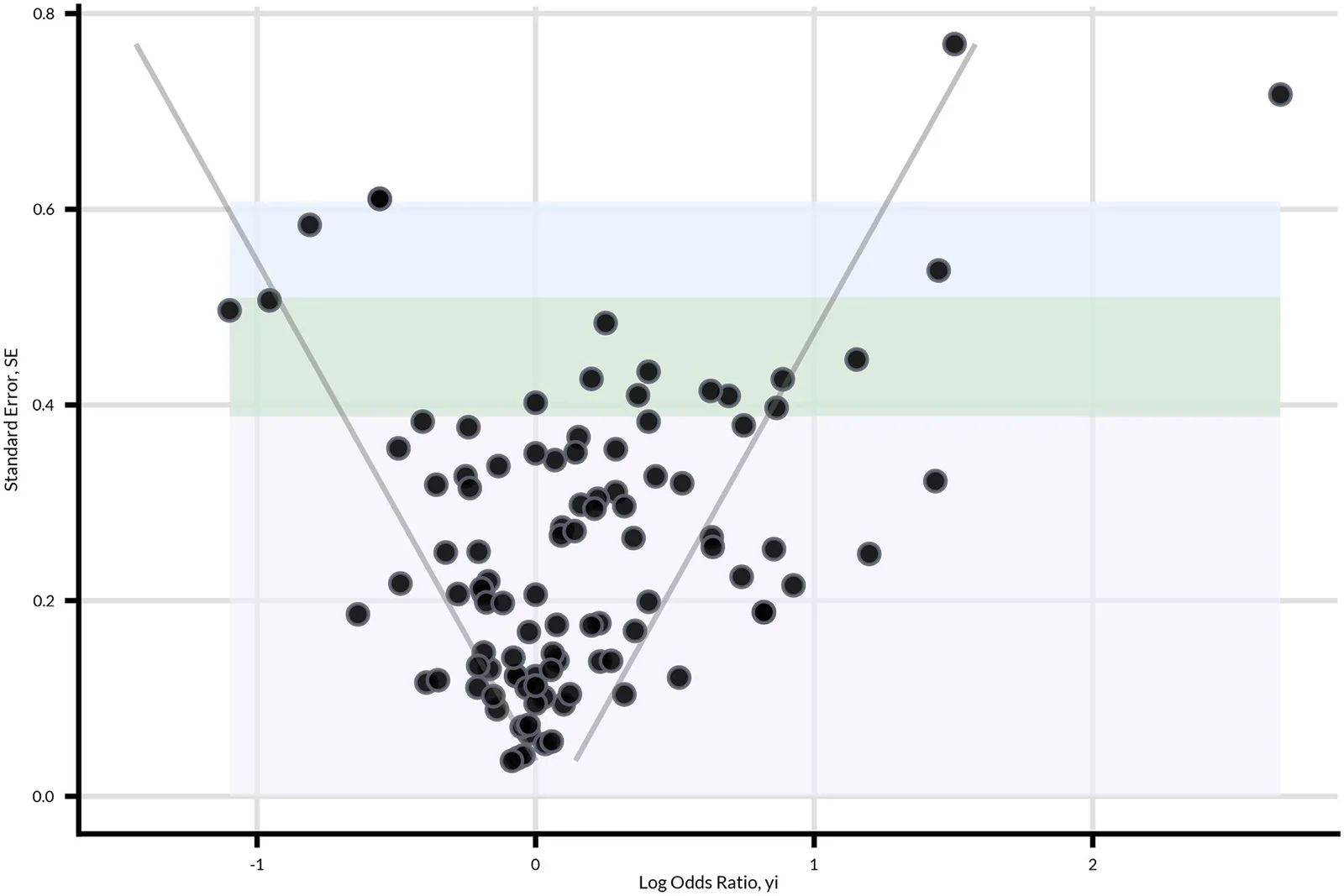
Contour-enhanced funnel plot of the multilevel meta-analysis. Individual effect sizes (log odds ratios; *n* = 103) are plotted against their standard errors. Shaded pastel contours correspond to conventional significance thresholds (*P* = 0.10, 0.05, 0.01). The diagonal funnel lines represent the expected 95% confidence region around the pooled effect sizes.

Before conducting moderator analyses, the heterogeneity was explored in several grouping variables (season, study type, study location, and risk factor) by fitting the MLMA model that included these factors as random effects. This variance partitioning revealed that publication-level differences accounted for the largest share of heterogeneity (σ2 = 0.0425), followed by season (σ2 = 0.0121), study location (σ2 = 0.0099), and entry-level variation (σ2 = 0.0097). These results suggest that certain variables may influence effect sizes, therefore they examined formally as moderators in subsequent models.

### Testing for moderators

Season-specific pooled effects were tested and showed great variation in BRD incidence, depending on the month cattle were received. March showed a significantly positive effect (ln*OR = *0.6840 ± 0.1835; *P* = 0.0002), corresponding to nearly twice the odds of cattle being healthy (OR = 1.98). Meanwhile, other months showed small (ln*OR* ≤ 0.2877) and non-significant effects (*P* ≥ 0.1072), with confidence intervals overlapping zero. A forest-style effect plot for each month in which cattle were received is shown in Fig. 3. The global moderator test indicated that season collectively explained significant variation within effect sizes (QM = 23.3007; *P* = 0.0253). Therefore, season may partially explain variation in treatment effects, with slightly greater effects observed in certain months.

**Figure 3:**
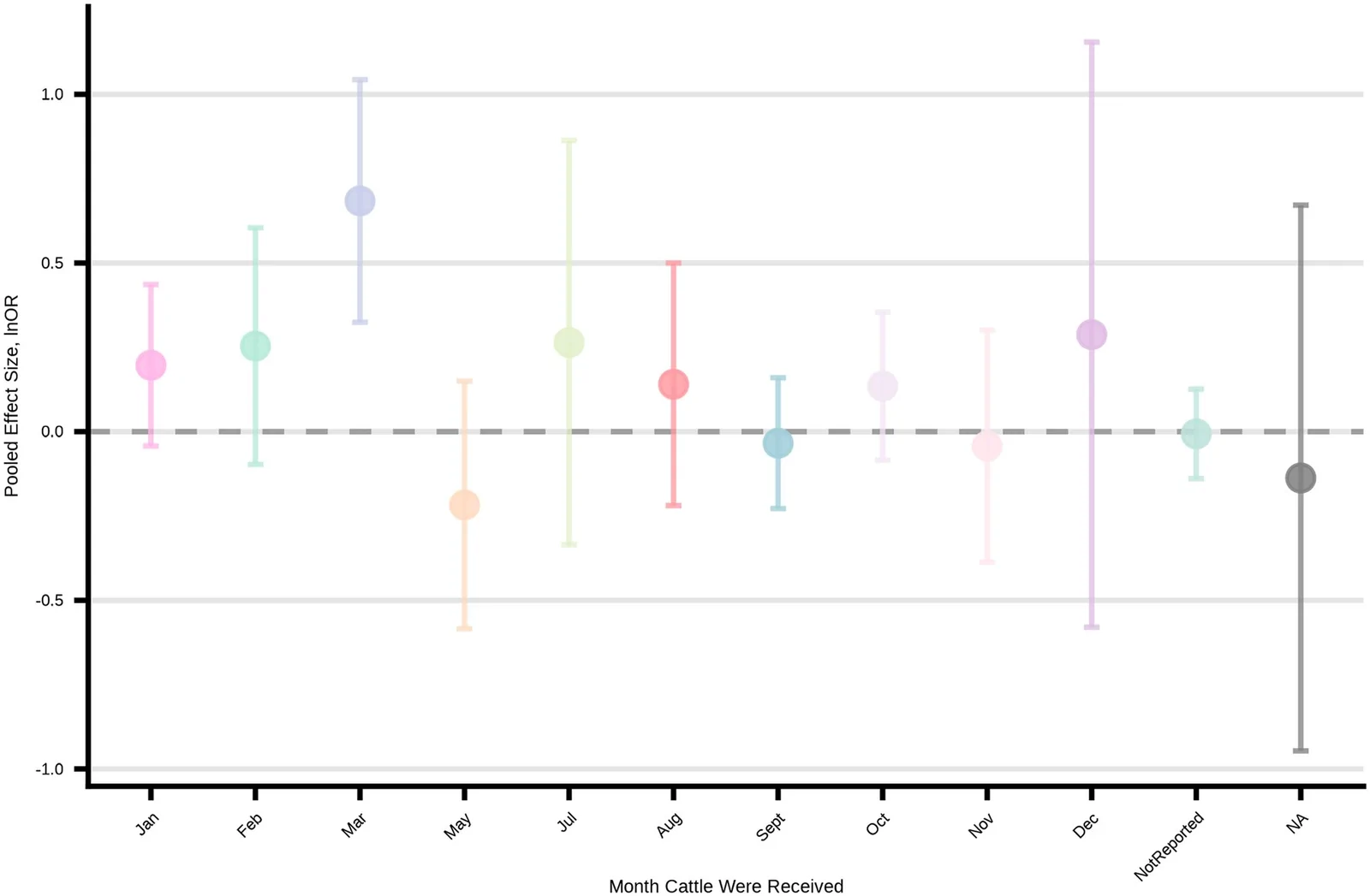
Season-specific pooled effect sizes (lnOR) for cattle health outcomes. Each point represents the estimates of effect size for the month in which cattle were received. Error bars indicate 95% confidence intervals. The dashed horizontal line at zero indicates no effect. Estimates were derived from multilevel random-effect meta-analysis accounting for study-level clustering.

There were a total of 5 interventions for BRD that were evaluated in this dataset, including metaphylactic therapy, nutritional intervention, new-product administration, technology diagnostics, and cattle sources. Metaphylactic programs resulted in a significantly positive effect (ln*OR = *0.1660 ± 0.0843; *P* = 0.0489), which corresponds to approximately 18% (OR = 1.18) greater odds of cattle remaining healthy. Studies that evaluated various technological diagnostic tools for BRD tended to have a positive effect (ln*OR = *0.3964 ± 0.2101; *P* = 0.0592) on BRD incidence. However, studies evaluating nutritional programs (ln*OR = *0.0209 ± 0.0635; *P* = 0.7422), various commercial products (ln*OR = *0.0424 ± 0.0996; *P* = 0.6701), and cattle deriving from numerous sources (ln*OR = *−0.0887 ± 0.2796; *P* = 0.7510) did not affect overall BRD incidence in this dataset. A forest-style effect plot for each BRD mitigation technique evaluated in this moderator test is shown in Fig. 4. Although metaphylactic studies showed significantly greater odds, study type overall did not explain between-study variation (QM = 7.8313; *P* = 0.1658).

**Figure 4:**
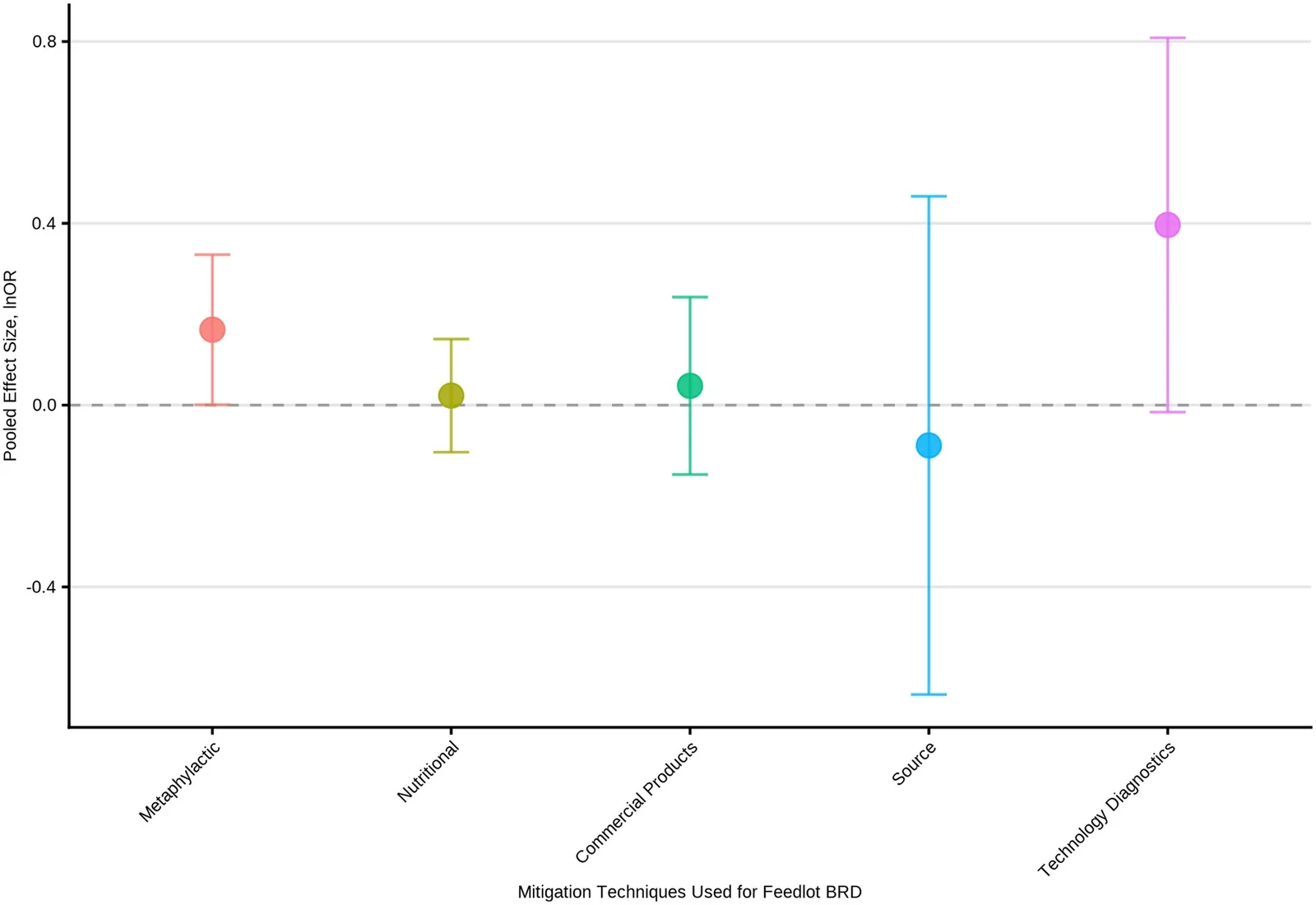
Mitigation technique-specific pooled effect sizes (lnOR) for cattle health outcomes. Each point represents the estimates of effect size for each type of BRD intervention evaluated. Error bars indicate 95% confidence intervals. The dashed horizontal line at zero indicates no effect. Estimates were derived from multilevel random-effect meta-analysis accounting for study-level clustering.

This dataset included research that was conducted in 17 different states within the United States, primarily the Southern and Midwest states. Studies that were conducted in Georgia (ln*OR = *0.7464 ± 0.2374; *P* = 0.0017) and Texas (ln*OR = *0.2501 ± 0.0806; *P* = 0.0019) had positive effects of significantly increasing the odds of cattle that remained healthy when BRD mitigation techniques were applied. However, all other locations showed no effect (ln*OR* ≤ 0.3567; *P* ≥ 0.1164) and were not significant. A forest-style effect plot for each location evaluated in this moderator test is shown in Fig. 5. Unlike the moderator test evaluating mitigation technique, location does explain significant between-study variation of effect sizes (QM = 32.08; *P* = 0.0147).

**Figure 5:**
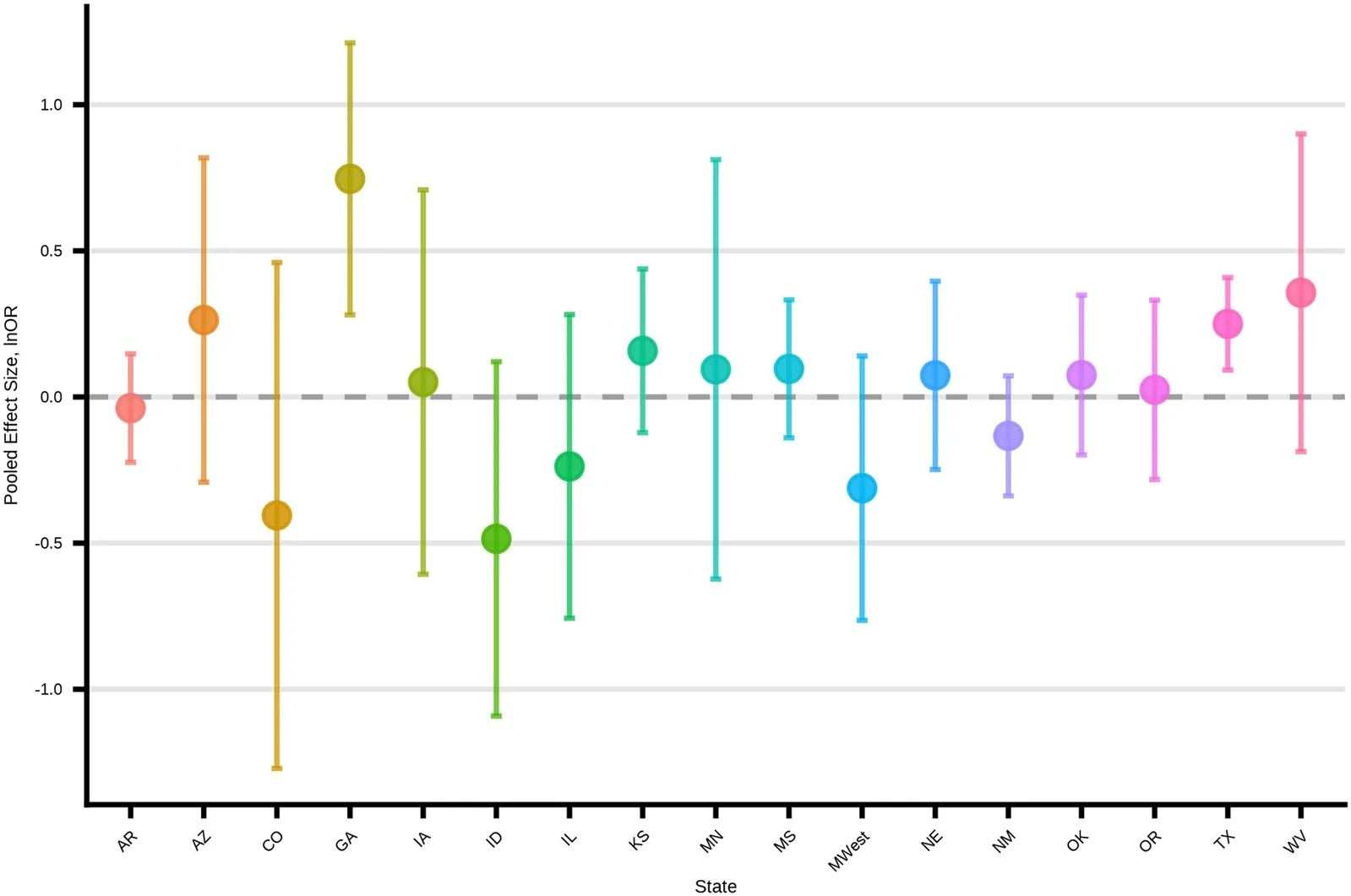
Location-specific pooled effect sizes (lnOR) for cattle health outcomes. Each point represents the estimates of effect size for each state that experiments were conducted. Error bars indicate 95% confidence intervals. The dashed horizontal line at zero indicates no effect. Estimates were derived from multilevel random-effect meta-analysis accounting for study-level clustering.

Based on the BRD risk-factors (LR or HR) that were assigned to each study within this dataset, there were a total of 91 data entries considered at high-risk for BRD and only 12 data entries considered at low-risk for BRD. Studies that were considered at HR (ln*OR = *0.0582 ± 0.0444; *P* = 0.1903) or LR (ln*OR = *0.2483 ± 0.1589; *P* = 0.1180) for BRD showed no effect on overall treatment morbidity outcomes. A forest-style effect plot for each risk-factor evaluated in this moderator test is shown in Fig. 6. Within this dataset, assigning a risk-factor based on study-level overall morbidity rates did not explain significant between-study variation of the reported effect sizes (QM = 4.16; *P* = 0.1250).

**Figure 6:**
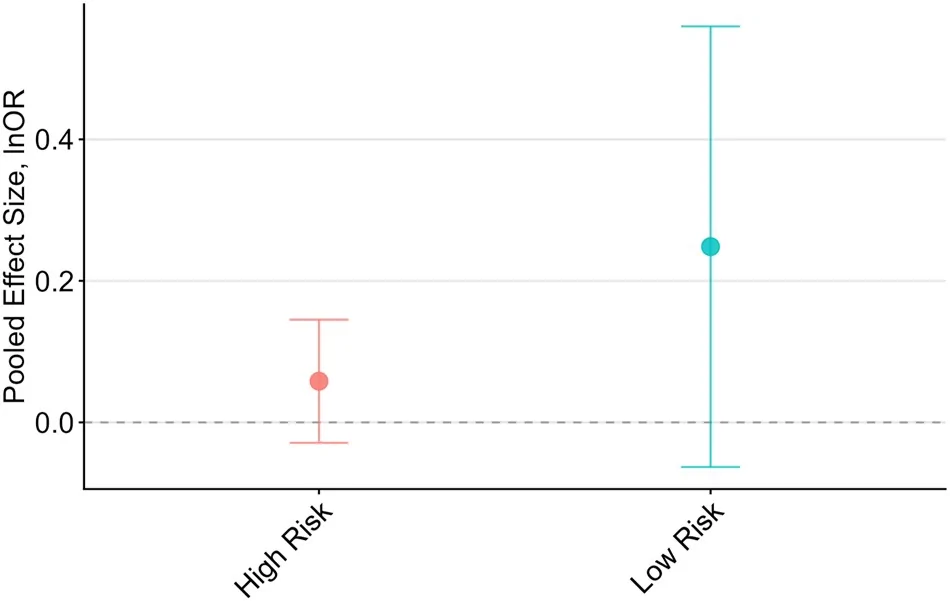
Risk factor-specific pooled effect sizes (lnOR) for cattle health outcomes. Each point represents the estimate of effect size for each risk factor assigned to the studies conducted. Each study was assigned 1 of 2 bovine respiratory disease (BRD) risk factors: (1) below 15% morbidity (LR = low risk), or (2) greater than or equal to 15% morbidity (HR = high risk). Risk factors were determined based on Rojas et al. (2022) recommendations. Error bars indicate 95% confidence intervals. The dashed horizontal line at zero indicates no effect. Estimates were derived from multilevel random-effect meta-analysis accounting for study-level clustering.

### Subset analysis

To investigate further, a subset analysis was performed to quantify the effects of nutritional (*n* = 47) and metaphylactic (*n* = 32) interventions evaluated specifically within the dataset.

This analysis resulted in 9 different nutritional mitigation techniques for BRD. Out of the 9 interventions that were evaluated, experiments testing vitamin E (ln*OR = *−0.3223; *P* = 0.0518) and pelleting feed (ln*OR = *0.3445; *P* = 0.0804) both were associated with greater BRD incidence. In turn, these results showed vitamin E (*n* = 5) having a negative effect and pelleted feed (*n* = 2) having a positive effect on BRD incidence, but it is important to note that the sample sizes were small for this analysis. Other nutrition-specific interventions, including trace mineral sources, roughage inclusion, medicated feed additives, immunomodulatory feed ingredients, and direct-fed microbials, showed no consistent effect on BRD (ln*OR* ≤ 0.0990) and were not significant (*P* ≥ 0.2377). An orchard-style effect plot for each nutritional intervention evaluated in this moderator test is shown in Fig. 7. Overall, these nutritional studies did not explain the between-study variation of BRD incidence (QM = 12.5081; *P* = 0.1862).

**Figure 7:**
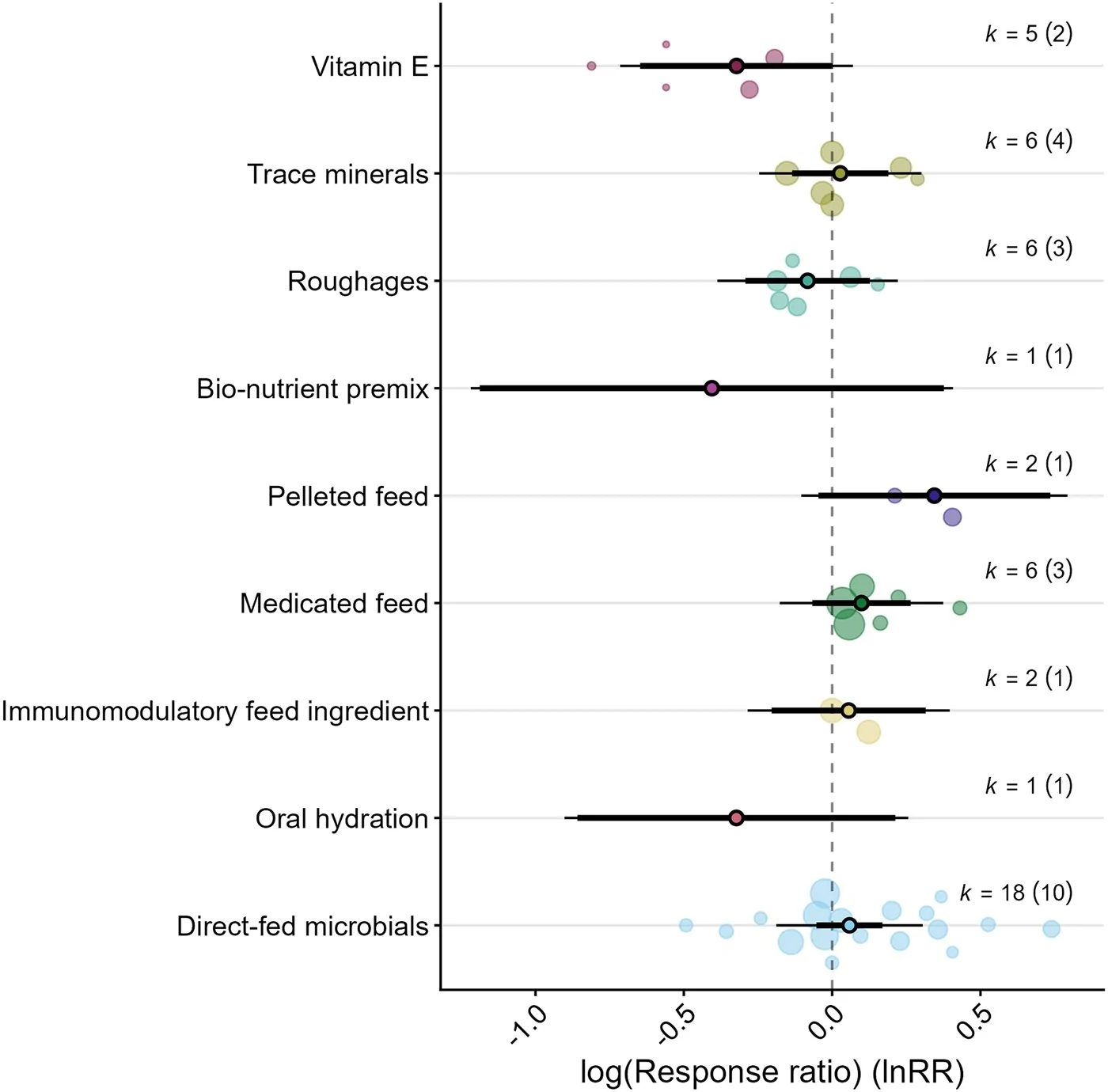
Nutrition-specific pooled effect sizes (lnOR) for cattle health outcomes. Each point represents the estimates of effect size for each type of nutritional mitigation evaluated. Error bars indicate 95% confidence intervals. The dashed horizontal line at zero indicates no effect. Estimates were derived from multilevel random-effect meta-analysis accounting for study-level clustering.

Furthermore, when testing study-level characteristics within studies evaluating metaphylactic programs, there were 3 total reported types of metaphylactic protocols tested to decrease BRD outcomes. Out of these, only the metaphylactic programs evaluating antimicrobial resistance decreased the odds of cattle being treated with antibiotics for BRD (lnOR = 1.2789; *P* = 0.0020). However, it is important to note that within the current dataset, there was only 1 study that investigated antimicrobial-resistance, so the effect size derived essentially from 1 study. Other metaphylactic experiments, testing the type of antimicrobial and timing of antimicrobial administration, showed no effect (ln*OR* ≥ 0.1840) and were not significant (*P* ≤ 0.2148). An orchard-style effect plot for each metaphylactic program evaluated in this moderator test is shown in Fig. 8. Overall, within this restricted dataset, metaphylactic programs significantly had a positive effect, driven primarily by the antimicrobial resistance experiments, which showed significantly greater odds of cattle remaining healthy (QM = 9.8630; *P* = 0.0198).

**Figure 8:**
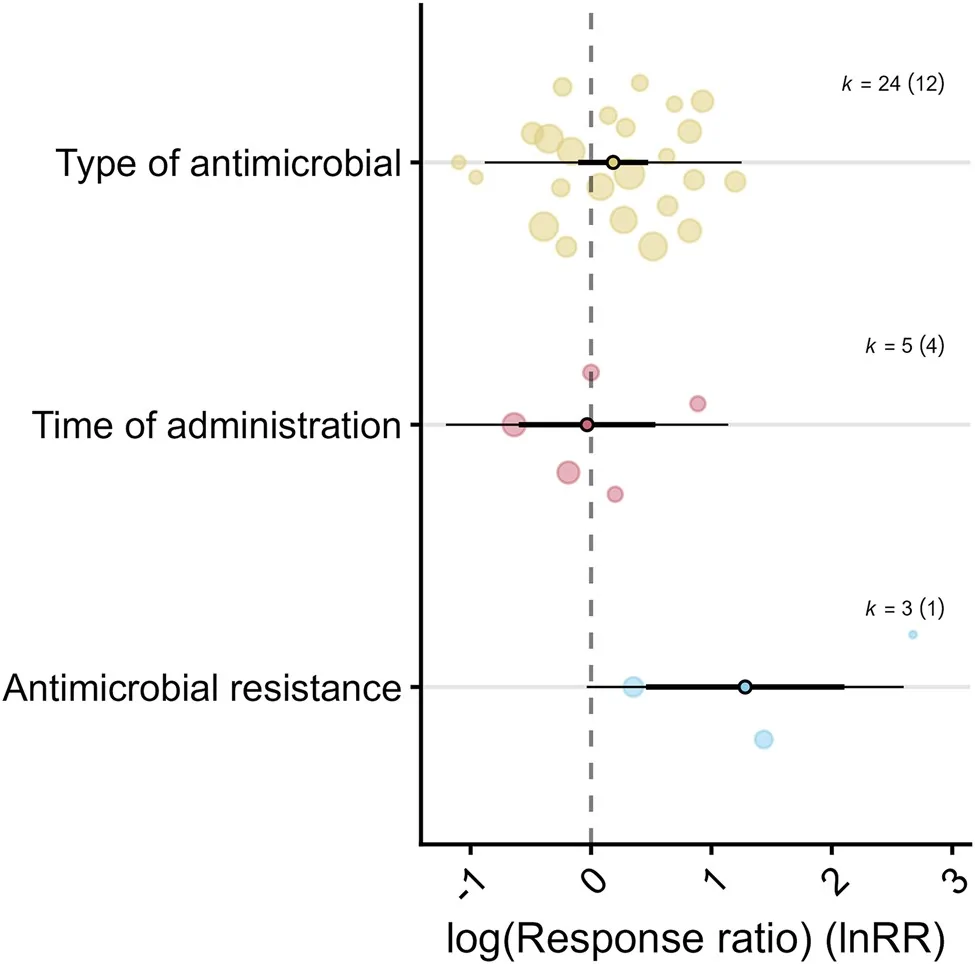
Metaphylactic-specific pooled effect sizes (lnOR) for cattle health outcomes. Each point represents the estimates of effect size for each type of metaphylactic program evaluated. Error bars indicate 95% confidence intervals. The dashed horizontal line at zero indicates no effect. Estimates were derived from multilevel random-effect meta-analysis accounting for study-level clustering.

### Residual heterogeneity

Residual heterogeneity was tested to identify the source of variation in the effect sizes that were produced among all studies. Study location, season, mitigation type, sex, and study sample size were all tested for variation. There was a significant presence of heterogeneity that remained across studies (*Q* = 335.9015; *P* < 0.001). Variance decomposition revealed that the greatest amount of heterogeneity was attributed to between-publication differences (*τ*^2^ = 0.0425), with smaller contributions from season (*τ*^2^ = 0.0121), location (*τ*^2^ = 0.0099), and within-publication clustering (*τ*^2^ = 0.0097). Additionally, no reported variance was attributed to study type, sex, or sample size.

### Publication bias

Potential small-study effects were evaluated using a multilevel Egger-type regression model that retained the hierarchical structure of effect sizes nested within publication. A significant association was observed between effect size and standard error (Fig. 9; *β* = 1.384, *P* < 0.0001), indicating evidence of funnel-plot asymmetry. However, the observed asymmetry should not be interpreted as definitive evidence of publication bias, as heterogeneity or other study-level differences may also contribute to this pattern.

**Figure 9:**
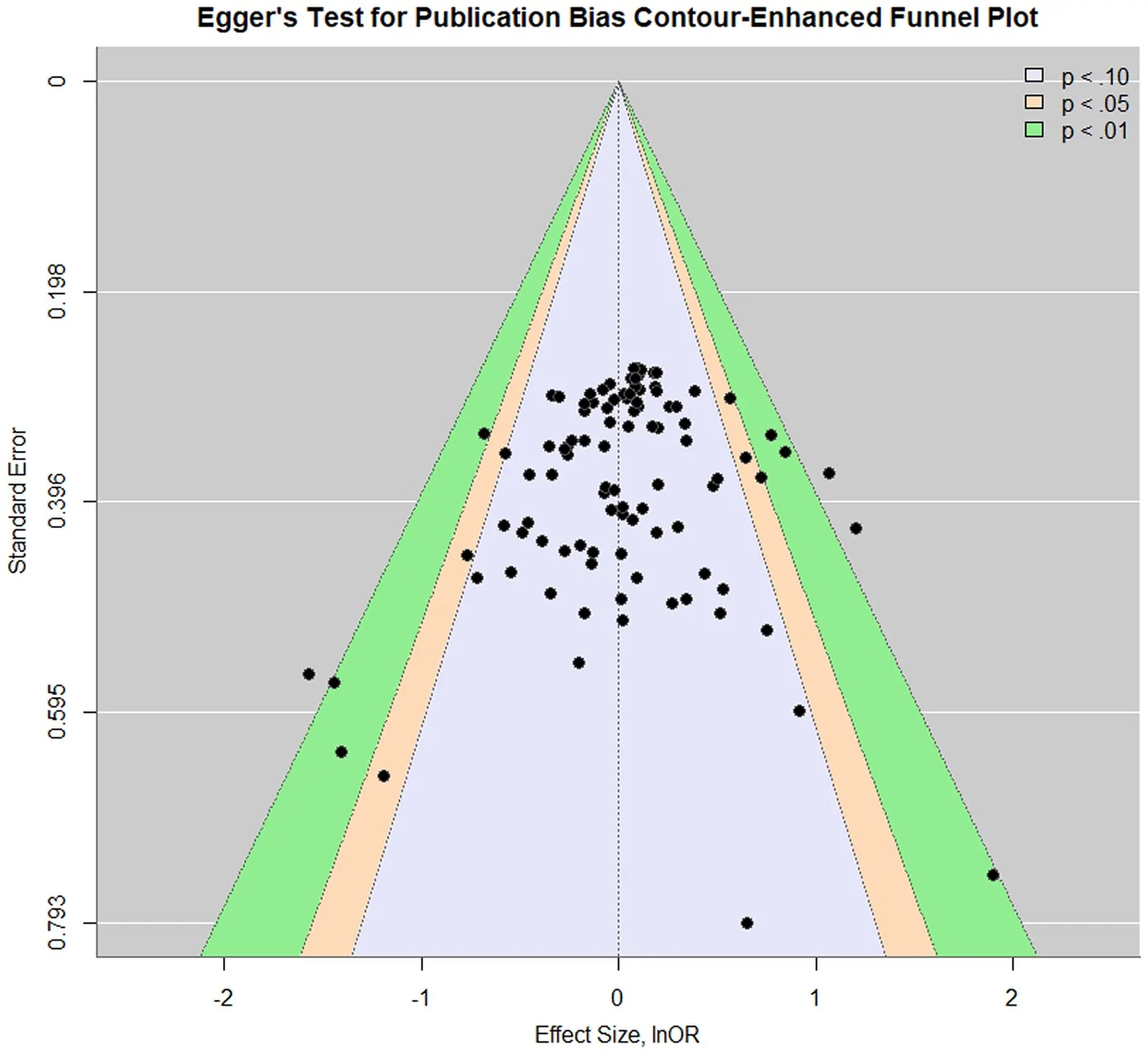
Contour-enhanced funnel plot showing Egger’s test for publication bias. Asymmetrical funnel plot indicates presence of publication bias. Black data points indicate effect sizes from each data entry. Egger’s test (*P* = 0.0016) and fail-safe number (74) indicate publication bias.

## Discussion

To our knowledge, this study provides the first meta-analysis examining all BRD interventions that have been implemented into literature over the previous decade in the United States. The meta-analysis conducted was a broad investigation that evaluated a variety of mitigation strategies for BRD, study locations, and different months in which beef calves were received. This study aimed to determine if experimental interventions for BRD have either increased or decreased the percentage of cattle that were treated at least once with antibiotics compared to control groups of cattle. It is important to note that the control groups of cattle in each study used for data entry met similar criteria and aligned with current protocols for “high-risk” cattle.

The conventional random-effects model yielded a small but statistically significant overall effect, meaning that overall, the BRD interventions evaluated did increase the odds of cattle remaining healthy. However, heterogeneity was substantial, indicating great variation in true effect sizes across studies. The multi-level random effects model was used to account for dependency among multiple effect sizes that were nested within publications. Across the 103 effect sizes calculated from 56 publications, the pooled effect suggests a modest increase in odds that cattle remained healthy when BRD mitigation strategies were applied. When accounting for the dependency of multiple effect sizes, using a multi-level model, the pooled estimate was attenuated and was no longer statistically significant, but remained a tendency. This suggests that part of the precision observed in the conventional model may reflect non-independence among effect sizes. Furthermore, the multi-level results provide a more conservative and arguably more accurate estimate of the overall influence that mitigation strategies had on BRD incidence.

As there was significant heterogeneity that remained between publications, variation was explored via moderator tests. Investigating study-level moderators assisted in explaining the driver(s) of the variance in effect sizes. The moderators that were tested in this analysis included mitigation-specific strategies (study type), the state where the experiment took place (location), and the month of the year in which cattle were received (season).

Study type showed modest differences, with metaphylactic programs demonstrating a small but statistically significant effect, whereas technology-based diagnostic strategies were borderline significant. Given its multifactorial nature, BRD requires a comprehensive approach for effective diagnosis and management, and the establishment of standardized diagnostic criteria would facilitate early intervention by enhancing the accuracy of diagnosis for BRD. Galyean et al. (2022) explain that flexibility is desired when setting cutoff points for treating BRD, and research is needed in newly received cattle to better define the options which is what the technology-based studies used in this meta-analysis evaluated. Studies evaluated in the current study tended to reduce BRD incidence, suggesting that utilizing technology-based diagnostic criteria for high-risk cattle in the receiving period could be beneficial.

One of the tools evaluated was an electronic stethoscope (Whisper), which has been developed to improve the interpretation of auscultation in cattle with BRD, and has been primarily adapted for use in feedlots. Nickell et al. (2021) evaluated the efficacy of the Whisper On Arrival system, which is patented and used chute-side to predict the risk of BRD in cattle at receiving. This rather new technology uses a sound-collection device to estimate heart and lung data, accompanied by manually entered rectal temperature and body weight data into a proprietary algorithm to determine whether to “treat” or “do not treat.” Nickell et al. (2021) reported a reduction in BRD with cattle that were treated based on the Whisper On Arrival diagnosis. Another tool used in the current study was a remote early disease identification (REDI) system that monitors cattle behavior to identify potential BRD cases. White et al. (2015) concluded that the REDI system was comparable to conventional management with potential advantages of an earlier BRD diagnosis and decreased use of antibiotics. Lastly, another tool used in the current study was the use of infrared thermography. Smock et al. (2023) reported a slight decrease in cattle treated for BRD when using diagnostic criteria from thermal imaging, however, overall morbidity percentages were considered low (>15%). Because the 3 different types of technology-based diagnostic tools did not report consistent reduction of BRD incidence, the difference revealed in this study was considered marginally significant.

Location, however, was a significant moderator, with Georgia and Texas showing greater effect sizes, suggesting regional variation may influence BRD incidence. It is important to point out that there was only 1 study conducted in Georgia, which was a metaphylactic study showing a significant reduction in BRD when administering tulathromycin. The month out of the year in which cattle were received was significant and assisted in accounting for some of the variation among effect sizes. Therefore, season may partially explain variation in treatment effects, with slightly greater effects observed in certain months. Season has been directly associated in previous studies with respiratory disease incidence and is likely a proxy for weather conditions, which vary by geographical location (Snowder et al. 2006; Terrell et al. 2011; Cernicchiaro et al. 2012; Denholm et al. 2024). It was concluded in a review studying the pathogenesis of BRD that although the basic features are relatively well understood, the multiple factors involved create complex interactions between the environment, infectious agents, and the host, which will continue to provide challenges to our understanding and management of BRD (Mosier 2015). Even though study location and season were both significant moderators in the current study, results only suggest that prioritizing management decisions by region and environment could assist in disease incidence.

The subset analysis that was conducted to investigate metaphylactic and nutritional experiments revealed a more detailed explanation for the variation that remained following study type moderator tests. Out of the 103 data entries (Table S1, available as supplementary data at *Translational Animal Science* online), 79 of them were either nutritional or metaphylactic interventions; it was important to break these 2 sections up for a more descriptive analysis.

Globally, the most common practice for preventing BRD in high-risk cattle at feedlot arrival is metaphylactic use of antimicrobials (Urban-Chmiel and Grooms 2012; Noyes et al. 2015). Metaphylactic use of antimicrobials serves as a method to proactively control the potential development and spread of BRD within a given group of cattle, regardless of whether only a subset of animals exhibits respiratory disease symptoms (Ives and Richeson 2015; Horton et al. 2023). In the current study, metaphylactic programs were associated with improved cattle health outcomes; however, this benefit must be considered alongside the antimicrobial resistance outcomes represented within this category. Notably, the improvement in cattle health observed with metaphylaxis coincided with increased multidrug resistance, demonstrating an important trade-off between short-term disease control and antimicrobial stewardship. Therefore, the efficacy of metaphylaxis in reducing BRD incidence should not be interpreted independently of its potential contribution to the selection and persistence of antimicrobial-resistant bacteria. There is great concern about antimicrobials used for metaphylactic use contributing to the development of antibiotic-resistant pathogens, which may even be transferred to humans. Saenz et al. (2004) conducted a study on antimicrobial resistance in *Escherichia coli* O157 from humans and animals, which showed that 23% of *E. coli* from cattle were resistant to at least 1 antimicrobial, and 71% were resistant to 2 or more antimicrobials. It is known that about 70% to 90% of antimicrobials administered to cattle are excreted intact or as metabolites via urine and feces (Masse et al. 2014). Even small amounts of antimicrobials have the potential to persist in the environment and contribute to the selection of resistant bacteria in the soil, which can spread through various pathways (Andersson and Hughes 2014).

When only looking at the studies evaluating metaphylactic programs (Table S1, available as supplementary data at *Translational Animal Science* online), there were greater data entries for the studies investigating the type of antimicrobials that were administered. Metaphylactic programs typically include the use of oxytetracycline, tilmicosin, florfenicol, tulathromycin, and tetracycline (Granados-Chinchilla and Rodriguez 2017). Even though the overall effect was not significant, it was positive and is worth investigating further. Programs that administered tulathromycin (Miller et al. 2016; Booker et al. 2017; Crosby et al. 2018, 2023; Theurer et al. 2018; Munoz et al. 2020; Coppin et al. 2022; Credille et al. 2024), tilmicosin (Hawley et al. 2016; Word et al. 2020), tildipirosin (Theurer et al. 2018; Nickell et al. 2020), and ceftiofur (Coppin et al. 2022) on the same day that cattle were received resulted in less cattle that were treated at least once for BRD. Studies that evaluated the time antibiotics were administered for metaphylactic use were also not significant in this analysis, but the studies that did decrease the odds of BRD incidence included administering antimicrobials on arrival and 17 days post-arrival (Celestino et al. 2020). Collectively, these findings demonstrate that metaphylactic use of antibiotics can provide short-term control of BRD under certain conditions; however, clinical efficacy alone does not capture the broader consequences of antimicrobial use. The concurrent emergence or selection of antimicrobial resistance emphasizes the need to balance immediate improvements in cattle health with long-term antimicrobial stewardship and supports continued development of effective non-antimicrobial BRD prevention strategies.

Nutritional mitigation of BRD, usually accompanied by an antibiotic protocol, is commonly used as a tool to prevent immunosuppression in high-risk cattle. Nutritional factors affecting BRD include energy, protein, roughage, water, minerals, vitamins, probiotics and prebiotics, and other additives. Nutritional interventions overall did not result in moderated effects due to the variation in BRD outcomes among the studies used. However, studies evaluating vitamin E and pelleting feed both result in significant effects. Administering injectable vitamin E to cattle before or after transit (Dornbach et al. 2023) and linearly increasing dietary levels of vitamin E (Deters and Hansen 2019) both increased the incidence of BRD within the receiving period. However, this dataset only included 2 studies testing whether Vitamin E could reduce BRD incidence, so the results should be interpreted cautiously. It was concluded in a review by Spears and Weiss (2014) that the immune function of feedlot cattle has been enhanced with supplemental vitamin E, but effects on morbidity and clinical health measurements have been small to nonexistent, which may explain the results in the current study.

Additionally, studies that evaluated pelleting corn residue (Peterson et al. 2015) decreased the incidence of BRD. When grains undergo grinding before pelleting, a reduction in the quantity of feed required per pound of grain has been observed previously (Roberts 1964). The experiments testing pelleted feed in the current study utilized corn residue to design a replacement for a conventional grain and forage diet with hopes of improving performance.

The greatest number of nutritional studies that were evaluated remained in the direct-fed microbials category. The overall effect of this moderator was not significant due to the variation in BRD incidence that was reported; it was slightly positive. There are several bacterial genera, species, and strains that have been listed as “Generally Recognized As Safe” for use in cattle rations, including *Lactobacillus* spp., *Megasphera* spp., *Propionibacterium* spp., and *Bacillus* spp. (Cappellozza et al. 2025). Direct-fed microbial supplementation that included *Bacillus subtilis* decreased BRD incidence (Smock et al. 2020; Idowu et al. 2022; Word et al. 2022; Ryan et al. 2023; Young et al. 2023). The inclusion of brewers’ yeast (Palmer et al. 2019) and yeast cell wall (Rivera et al. 2019) increased BRD incidence. In agreement, *Bacillus* spp. has been shown to support the health of the host, competitive exclusion against potentially harmful bacteria, promoting synthesis and release of antimicrobial and antioxidant compounds, and maintaining the integrity of intestinal epithelial cells (Cappellozza et al. 2025). Recent work has demonstrated that *Bacillus*-based direct-fed microbials can enhance health (Mackey et al. 2024) and feed efficiency (Dias et al. 2022; Cordeiro et al. 2024) in growing cattle.

Although several moderators significantly explained variability in effect sizes, substantial residual heterogeneity remained. Given this variability and the modest magnitude of the pooled effect result, the potential influence of publication bias and small-study effects was evaluated to assess the robustness of the findings.

The test of small-study effects indicated potential asymmetry. Asymmetry can be driven by publication bias, true heterogeneity, study quality differences, or outcome reporting bias. The Egger’s regression test was significant, suggesting that smaller studies may report larger effects. This pattern raises the possibility of publication bias or other sources of asymmetry, such as true heterogeneity or methodological differences across studies. To further assess robustness, a fail-safe N analysis indicated that 74 additional null studies would be required to render the overall effect of non-significance. While this suggests moderate robustness, the adjusted effect size under this scenario was substantially attenuated. Furthermore, the pooled effect was sensitive to model specification, becoming non-significant when dependency among effect sizes was accounted for in the multilevel model. It is important to note that the current study only utilizes 1 database to search for relevant literature, which could be a limiting factor of the study. Taken together, these findings suggest that although the association is observable, it is modest in magnitude and sensitive to both modeling assumptions and small-study effects.

## Conclusion

To conclude, the incidence of BRD tended to decrease in cattle that were applied with treatments, but there was great variation among studies; therefore, the magnitude varied. The results suggest prioritizing management decisions based on environmental and regional conditions. Interventions for BRD in this analysis suggest that metaphylactic programs and technological diagnostics may be more effective in certain contexts. Although moderators were tested in the multi-level model, variability in effect sizes were unexplained. These findings serve to direct future research toward alternative approaches that may reduce antimicrobial use without compromising control of respiratory infection outbreaks.

## Supplementary Material

txag152_Supplementary_Data

## List of abbreviations

BRD: Bovine respiratory disease
PRISMA: Preferred reporting items for systematic review and meta-analyses
MLMA: Multi-level meta-analytical model
LR: Low risk
HR: High risk
