# Supplementary material for: Seasonality of bovine respiratory disease and mitigation techniques used in receiving beef cattle in the last 10 years: a meta-analysis
Source: Transl Anim Sci. 2026 Oct 5;10:txag152. doi: 10.1093/tas/txag152 (PMC13662695; doi:10.1093/tas/txag152)
Supplement: Supplementary file 1 [file txag152_supplementary_data.docx]

**Supplementary Table 1.** Raw data and variables available for calculations included for meta-analysis.

|  |  |  |  | **Control^3^** | | **Treatment^4^** | | **Variables** | | | | | |
| --- | --- | --- | --- | --- | --- | --- | --- | --- | --- | --- | --- | --- | --- |
| **Entry** | **Publication** | **State^1^** | **n^2^** | **BRD, %** | **Healthy, %** | **BRD, %** | **Healthy, %** | **N^5^** | **Season^6^** | **Sex^7^** | **Type^8^** | **Exp^9^** | **Risk^10^** |
| 1 | Martin et al. 2020 | KS | 2 | 27 | 73 | 32 | 68 | 384 | Oct | SB | Product | NSAID | HR |
| 2 | Miller et al. 2016 | NM | 3 | 5 | 95 | 15 | 85 | 386 | NA | H | Metaphylactic | Type | HR |
| 3 | Miller et al. 2016 | NM | 3 | 5 | 95 | 13 | 87 | 386 | NA | H | Metaphylactic | Type | HR |
| 4 | Robison et al. 2023 | OK | 3 | 14 | 86 | 16 | 84 | 393 | April | H | Nutritional | Roughage | HR |
| 5 | Robison et al. 2023 | OK | 3 | 14 | 86 | 12 | 88 | 393 | Aug | H | Nutritional | Roughage | HR |
| 6 | Booker et al. 2017 | ID | 2 | 24 | 76 | 39 | 61 | 563 | NA | SH | Metaphylactic | Type | HR |
| 7 | Hawley et al. 2016 | AR | 2 | 20 | 80 | 15 | 85 | 202 | NA | SB | Metaphylactic | Type | HR |
| 8 | Word et al. 2022 | TX | 2 | 13 | 87 | 9 | 91 | 2340 | Jan | S | Nutritional | DFM | LR |
| 9 | Wells 2016 | IA | 2 | 17 | 83 | 4 | 96 | 772 | Aug | SH | Product | Vaccine | LR |
| 10 | Young et al. 2023 | TX | 6 | 22 | 78 | 16 | 84 | 480 | Mar | S | Nutritional | DFM | HR |
| 11 | Young et al. 2023 | TX | 6 | 22 | 78 | 13 | 87 | 480 | Mar | S | Nutritional | DFM | HR |
| 12 | Young et al. 2023 | TX | 6 | 22 | 78 | 20 | 80 | 480 | Mar | S | Nutritional | DFM | HR |
| 13 | Ryan et al. 2023 | OK | 2 | 12 | 88 | 8 | 92 | 397 | Sept | S | Nutritional | DFM | LR |
| 14 | McNeff et al. 2023 | OK | 2 | 15 | 85 | 14 | 86 | 406 | Oct | SBH | Product | Vaccine | LR |
| 15 | Celestino et al. 2020 | MN | 3 | 11 | 89 | 11 | 89 | 1400 | Jan | H | Metaphylactic | Timing | HR |
| 16 | Celestino et al. 2020 | MN | 3 | 11 | 89 | 9 | 91 | 1400 | Jan | H | Metaphylactic | Timing | HR |
| 17 | Crosby et al. 2023 | MS | 2 | 22 | 78 | 27 | 73 | 82 | Oct | H | Metaphylactic | Type | HR |
| 18 | Crosby et al. 2023 | MS | 2 | 27 | 73 | 19 | 81 | 83 | Oct | H | Metaphylactic | Resistance | HR |
| 19 | Crosby et al. 2023 | MS | 2 | 29 | 71 | 2 | 98 | 84 | Oct | H | Metaphylactic | Resistance | HR |
| 20 | Crosby et al. 2023 | MS | 2 | 42 | 58 | 10 | 90 | 82 | Mar | H | Metaphylactic | Resistance | HR |
| 21 | Theurer et al. 2018 | KS | 2 | 15 | 85 | 13 | 87 | 593 | Nov | SBH | Metaphylactic | Type | HR |
| 22 | Theurer et al. 2018 | KS | 4 | 14 | 86 | 18 | 82 | 295 | Nov | SBH | Metaphylactic | Type | HR |
| 23 | Theurer et al. 2018 | KS | 4 | 16 | 84 | 8 | 92 | 298 | Nov | SBH | Metaphylactic | Type | HR |
| 24 | Nickell et al. 2020 | NE | 6 | 15 | 85 | 19 | 81 | 1190 | Feb | S | Metaphylactic | Type | HR |
| 25 | Nickell et al. 2020 | KS | 6 | 15 | 85 | 10 | 90 | 1190 | Feb | S | Metaphylactic | Type | HR |
| 26 | Nickell et al. 2020 | KS | 6 | 15 | 85 | 8 | 92 | 1190 | Feb | S | Metaphylactic | Type | HR |
| 27 | Pollreisz et al. 2022 | NE | 2 | 29 | 71 | 35 | 65 | 1000 | Nov | H | Metaphylactic | Timing | HR |
| 28 | Coetzee et al. 2020 | MWest | 6 | 50 | 50 | 59 | 41 | 1490 | NA | SBH | Metaphylactic | Type | HR |
| 29 | Coetzee et al. 2020 | MWest | 6 | 50 | 50 | 74 | 26 | 1490 | NA | SBH | Metaphylactic | Type | HR |
| 30 | Coetzee et al. 2020 | MWest | 6 | 50 | 50 | 71 | 29 | 1490 | NA | SBH | Metaphylactic | Type | HR |
| 31 | Schumaher et al. 2018 | OR | 3 | 32 | 68 | 17 | 83 | 106 | NA | SH | Product | Vaccine | HR |
| 32 | Schumaher et al. 2018 | OR | 3 | 32 | 68 | 32 | 68 | 106 | NA | SH | Product | Vaccine | HR |
| 33 | Nickell et al. 2021 | TX | 6 | 19 | 81 | 6 | 94 | 2560 | NA | S | Technology | Whisper | HR |
| 34 | Nickell et al. 2021 | NE | 6 | 19 | 81 | 8 | 92 | 2560 | NA | S | Technology | Whisper | HR |
| 35 | Nickell et al. 2021 | OK | 6 | 19 | 81 | 9 | 91 | 2560 | NA | S | Technology | Whisper | HR |
| 36 | Dornbach et al. 2023 | IL | 3 | 28 | 72 | 34 | 66 | 130 | NA | S | Nutritional | Vit E | HR |
| 37 | Dornbach et al. 2023 | IL | 3 | 28 | 72 | 37 | 63 | 130 | NA | S | Nutritional | Vit E | HR |
| 38 | Pickett et al. 2024 | TX | 2 | 57 | 43 | 57 | 43 | 120 | Sept | S | Product | MBAS | HR |
| 39 | Silva et al. 2018 | OR | 3 | 11 | 89 | 14 | 86 | 56 | Sept | S | Nutritional | DFM | HR |
| 40 | Silva et al. 2018 | OR | 3 | 11 | 89 | 18 | 82 | 56 | Sept | S | Nutritional | DFM | HR |
| 41 | Rivera et al. 2019 | MS | 2 | 82 | 18 | 84 | 16 | 240 | Sept | SH | Nutritional | DFM | HR |
| 42 | Woolsoncroft et al. 2018 | OK | 4 | 31 | 69 | 37 | 63 | 94 | Aug | S | Nutritional | Roughage | HR |
| 43 | Woolsoncroft et al. 2018 | OK | 4 | 32 | 68 | 36 | 64 | 94 | Aug | S | Nutritional | Roughage | HR |
| 44 | CREDIlle et al. 2023 | GA | 2 | 40 | 60 | 17 | 83 | 155 | Feb | SBH | Metaphylactic | Type | HR |
| 45 | Szasz et al. 2018 | AZ | 4 | 20 | 80 | 13 | 87 | 3400 | Jul | S | Nutritional | Medicated | HR |
| 46 | Szasz et al. 2018 | AZ | 4 | 20 | 80 | 16 | 84 | 3400 | Jul | S | Nutritional | Medicated | HR |
| 47 | Szasz et al. 2018 | AZ | 4 | 20 | 80 | 17 | 83 | 3400 | Jul | S | Nutritional | Medicated | HR |
| 48 | Crosby et al. 2018 | GA | 2 | 34 | 66 | 18 | 82 | 341 | Mar | SB | Metaphylactic | Type | HR |
| 49 | Powledge et al. 2022 | TX | 3 | 55 | 45 | 59 | 41 | 350 | Nov | SB | Product | Vaccine | HR |
| 50 | Powledge et al. 2022 | TX | 3 | 55 | 45 | 59 | 41 | 350 | Nov | SB | Product | Vaccine | HR |
| 51 | Rivera et al. 2018 | MS | 2 | 73 | 27 | 66 | 34 | 320 | NA | H | Nutritional | Medicated | HR |
| 52 | Nelson et al. 2022 | CO | 2 | 10 | 90 | 15 | 85 | 7678 | May | SH | Nutritional | Premix | LR |
| 53 | Peterson et al. 2015 | NE | 4 | 21 | 79 | 17 | 83 | 659 | Oct | S | Nutritional | Pelleted | HR |
| 54 | Peterson et al. 2015 | NE | 4 | 42 | 58 | 28 | 72 | 659 | Oct | S | Nutritional | Pelleted | HR |
| 55 | Cheek et al. 2024 | AR | 2 | 58 | 42 | 46 | 54 | 287 | Oct | H | Nutritional | TM | HR |
| 56 | Deters and Hansen 2019 | IA | 4 | 4 | 96 | 9 | 91 | 110 | Oct | S | Nutritional | Vit E | LR |
| 57 | Deters and Hansen 2019 | IA | 4 | 4 | 96 | 7 | 93 | 110 | Oct | S | Nutritional | Vit E | LR |
| 58 | Deters and Hansen 2019 | IA | 4 | 4 | 96 | 7 | 93 | 110 | Oct | S | Nutritional | Vit E | LR |
| 59 | Richeson et al. 2015 | AR | 4 | 90 | 10 | 96 | 4 | 101 | Sept | SB | Product | Implant | HR |
| 60 | Richeson et al. 2015 | AR | 4 | 90 | 10 | 94 | 6 | 102 | Sept | SB | Product | Implant | HR |
| 61 | Richeson et al. 2015 | AR | 4 | 90 | 10 | 98 | 2 | 101 | Sept | SB | Product | Implant | HR |
| 62 | Ryan et al. 2015 | AR | 3 | 61 | 39 | 61 | 39 | 175 | Feb | SBH | Nutritional | TM | HR |
| 63 | Ryan et al. 2015 | AR | 3 | 61 | 39 | 63 | 37 | 175 | Feb | SBH | Nutritional | TM | HR |
| 64 | Palmer et al. 2019 | AR | 3 | 67 | 33 | 77 | 23 | 87 | May | SB | Nutritional | DFM | HR |
| 65 | Palmer et al. 2019 | AR | 3 | 67 | 33 | 65 | 35 | 86 | Sept | SB | Nutritional | DFM | HR |
| 66 | Word et al. 2020 | TX | 3 | 77 | 23 | 56 | 44 | 99 | NA | SB | Metaphylactic | Type | HR |
| 67 | Word et al. 2020 | TX | 3 | 77 | 23 | 46 | 54 | 99 | NA | SB | Metaphylactic | Type | HR |
| 68 | White et al. 2015 | KS | 2 | 48 | 52 | 52 | 48 | 746 | NA | SBH | Technology | REDI | HR |
| 69 | Roberts et al. 2016 | TX | 2 | 16 | 84 | 12 | 88 | 128 | Dec | SB | Product | TM | LR |
| 70 | Munoz et al. 2020 | TX | 4 | 53 | 47 | 21 | 79 | 239 | Jan | SB | Metaphylactic | Type | HR |
| 71 | Munoz et al. 2020 | TX | 4 | 53 | 47 | 49 | 51 | 239 | Jan | SB | Metaphylactic | Type | HR |
| 72 | Munoz et al. 2020 | TX | 4 | 53 | 47 | 16 | 84 | 239 | Jan | SB | Metaphylactic | Type | HR |
| 73 | Homolka et al. 2023 | TX | 3 | 14 | 86 | 20 | 80 | 172 | NA | S | Nutritional | DFM | HR |
| 74 | Homolka et al. 2023 | TX | 3 | 14 | 86 | 14 | 86 | 172 | NA | S | Nutritional | DFM | HR |
| 75 | Galyen et al. 2020 | AR | 3 | 89 | 11 | 86 | 14 | 265 | Sept | SB | Nutritional | Medicated | HR |
| 76 | Galyen et al. 2020 | AR | 3 | 89 | 11 | 84 | 16 | 265 | Jan | SB | Nutritional | Medicated | HR |
| 77 | Ball et al. 2019 | AR | 2 | 28 | 72 | 53 | 47 | 176 | Sept | SB | Metaphylactic | Timing | HR |
| 78 | Horton et al. 2023 | KS | 2 | 17 | 83 | 7 | 93 | 2366 | Oct | SH | Metaphylactic | Timing | LR |
| 79 | Coppin et al. 2022 | TX | 4 | 59 | 41 | 26 | 74 | 238 | Oct | S | Metaphylactic | Type | HR |
| 80 | Coppin et al. 2022 | TX | 4 | 59 | 41 | 26 | 74 | 238 | Oct | S | Metaphylactic | Type | HR |
| 81 | Coppin et al. 2022 | TX | 4 | 59 | 41 | 45 | 55 | 238 | Oct | S | Metaphylactic | Type | HR |
| 82 | Lippolis et al. 2017 | OR | 3 | 69 | 31 | 61 | 39 | 72 | NA | S | Nutritional | IMFI | HR |
| 83 | Lippolis et al. 2017 | OR | 3 | 69 | 31 | 69 | 31 | 72 | NA | S | Nutritional | IMFI | HR |
| 84 | Gouvea et al. 2023 | NM | 4 | 50 | 50 | 47 | 53 | 72 | NA | S | Nutritional | Roughage | HR |
| 85 | Gouvea et al. 2023 | NM | 4 | 44 | 56 | 53 | 47 | 72 | NA | S | Nutritional | Roughage | HR |
| 86 | Smock et al. 2023 | TX | 4 | 9 | 91 | 2 | 98 | 120 | Mar | S | Technology | IRTI | LR |
| 87 | Smock et al. 2023 | TX | 4 | 9 | 91 | 7 | 93 | 120 | Mar | S | Technology | IRTI | LR |
| 88 | Wiegand et al. 2020 | NM | 3 | 56 | 44 | 53 | 47 | 96 | NA | H | Source | Commingling | HR |
| 89 | Wiegand et al. 2020 | NM | 3 | 56 | 44 | 69 | 31 | 96 | NA | H | Source | Commingling | HR |
| 90 | Colombo et al. 2020 | NM | 2 | 48 | 52 | 59 | 41 | 342 | NA | S | Product | MBAS | HR |
| 91 | Colombo et al. 2021 | NM | 3 | 78 | 22 | 82 | 18 | 170 | NA | S | Nutritional | DFM | HR |
| 92 | Colombo et al. 2021 | NM | 3 | 78 | 22 | 80 | 20 | 171 | NA | S | Nutritional | DFM | HR |
| 93 | Tomczak et al. 2019 | TX | 2 | 21 | 79 | 29 | 71 | 664 | Oct | H | Nutritional | Hydration | HR |
| 94 | Idowu et al. 2022 | WV | 2 | 50 | 50 | 35 | 65 | 80 | NA | S | Nutritional | DFM | HR |
| 95 | Cordero et al. 2024 | MS | 3 | 61 | 39 | 71 | 29 | 120 | NA | S | Nutritional | TM | HR |
| 96 | Cordero et al. 2024 | MS | 3 | 61 | 39 | 61 | 39 | 120 | NA | S | Nutritional | TM | HR |
| 97 | Duff et al. 2019 | NM | 3 | 41 | 59 | 38 | 62 | 120 | NA | SB | Product | Vaccine | HR |
| 98 | Duff et al. 2019 | NM | 3 | 41 | 59 | 42 | 58 | 120 | NA | SB | Product | Vaccine | HR |
| 99 | Van Engen et al. 2019 | OK | 3 | 23 | 77 | 21 | 79 | 112 | Aug | S | Product | Meloxicam | HR |
| 100 | Van Engen et al. 2019 | OK | 3 | 23 | 77 | 20 | 80 | 112 | Aug | S | Product | Meloxicam | HR |
| 101 | Smock et al. 2020 | TX | 4 | 44 | 56 | 35 | 65 | 192 | Jan | SB | Nutritional | DFM | HR |
| 102 | Smock et al. 2020 | TX | 4 | 44 | 56 | 36 | 64 | 192 | Jan | SB | Nutritional | DFM | HR |
| 103 | Smock et al. 2020 | TX | 4 | 44 | 56 | 21 | 79 | 192 | Jan | SB | Nutritional | DFM | HR |

^1^State where the study took place within the United States.

^2^The number of experimental treatments within the study.

^3^The percentage of cattle on the control treatments that were not treated (healthy) or treated (BRD) at least once with antibiotics for BRD.

^4^The percentage of cattle on mitigation treatments that were not treated (healthy) or treated (BRD) at least once with antibiotics for BRD.

^5^The number of calves included in the study.

^6^The calendar month in which cattle were received.

^7^The sex of the calves used in the study. S = steers, H = heifers, B = bulls, SB = steers and bulls, SH = steers and heifers, SBH = a mix of steers, bulls, and heifers.

^8^Type of BRD mitigation technique applied to the treatment group of calves.

^9^Detailed description of BRD mitigation technique applied to the treatment group of calves. NSAID = nonsteroidal anti-inflammatory drug, DFM = direct-fed microbial, MBAS = maternal bovine appeasing substance, TM = trace minerals, IMFI = immunomodulatory feed ingredient.

^10^Overall morbidity percentages associated with BRD were calculated [(total number of cattle treated for BRD / total number of cattle on trial) × 100] for each study and included to the dataset by adding a new column. Each study was then assigned 1 of 2 risk factors: 1) below 15% morbidity (LR = low risk), or 2) greater than or equal to 15% morbidity (HR = high risk). Risk factors were determined based on Rojas et al. (2022) recommendations.

**Supplementary Table 2.** List of literature cited and used to extract raw data and variables available for calculations included for meta-analysis.

| **Entry number** | **Citation** |
| --- | --- |
| 1 | Martin, M., M. Kleinhenz, K. Kleinhenz, E. Reppert, D. Blasi, T. Parks, A. Baysinger, J. Hutcheson, and J. Coetzee. 2020. Comparison of the effect of tildipirosin administered alone or in combination with transdermal flunixin on the performance, health, activity, and well-being of transported feedlot calves on arrival at the feedlot. Transl. Anim. Sci. 4. <https://doi.org/10.1093/TAS/TXAA005>. |
| 2, 3 | Miller, T. J., M. E. Hubbert, C. D. Reinhardt, C. A. Löest, E. F. Schwandt, and D. U. Thomson. 2016. Comparison of tulathromycin, tilmicosin, and gamithromycin for metaphylactic treatment of high-risk calves for control of bovine respiratory disease. Bov. Pract. (Stillwater). <https://doi.org/10.21423/bovine-vol50no2p175-179>. |
| 4, 5 | Robison, C. A., K. N. Pierce, R. R. Reuter, A. L. Warner, and B. K. Wilson. 2023. The effects of receiving diet roughage inclusion on performance, health, and serum metabolite characteristics of newly received beef calves. Transl. Anim. Sci. 7. <https://doi.org/10.1093/tas/txad039>. |
| 6 | Booker, C. W., T. Perrett, R. K. Fenton, E. J. Behlke, S. J. Hannon, B. K. Wildman, R. D. Rademacher, C. A. McMullen, B. D. Hunsaker, T. Hill, and G. K. Jim. 2017. An evaluation of concomitant therapy for the treatment of arrival fever in feedlot calves at ultra-high risk of developing undifferentiated fever/bovine respiratory disease. Int. J. Appl. Res. Vet. Med. 15. |
| 7 | Hawley, J., J. G. Powell, E. B. Kegley, and P. A. Beck. 2016. Comparison of ceftiofur crystalline free acid to tilmicosin for metaphylactic treatment of calves at risk for bovine respiratory disease. Bov. Pract. (Stillwater). <https://doi.org/10.21423/bovine-vol50no1p33-39>. |
| 8 | Word, A. B., T. A. Wickersham, L. A. Trubenbach, G. B. Mays, and J. E. Sawyer. 2020. Effects of metaphylaxis on production responses and total antimicrobial use in high-risk beef calves. App. Anim. Sci. 36. <https://doi.org/10.15232/aas.2019-01914>. |
| 9 | Wells, M. R. 2015. Comparing respiratory disease treatment rates in preconditioned calves vaccinated with 4-way killed or modified-live virus vaccine. Bov. Pract. (Stillwater). <https://doi.org/10.21423/bovine-vol49no2p132-139>. |
| 10, 11, 12 | Young, J. D., A. Waite, V. Nenov, N. Salah, and J. T. Richeson. 2023. Effects of Saccharomyces cerevisiae-derived yeast fraction and multispecie Bacillus supplementation on health and performance outcomes of newly received feedlot steers. App. Anim. Sci. 39. <https://doi.org/10.15232/aas.2023-02402>. |
| 13 | Ryan, W. R., E. S. DeSocio, M. E. Youngers, C. G. Lockard, C. J. Richards, S. J. Trojan, J. E. Hergenreder, and B. K. Wilson. 2023. Effects of feeding CLOSTAT (Bacillus subtilis PB6) on the clinical health, performance, and carcass characteristics of feedlot steers. Transl. Anim Sci. 7. <https://doi.org/10.1093/tas/txad047>. |
| 14 | McNeff, C. A., C. A. Robison, B. K. Wilson, F. J. White, R. Cauble, R. Biggs, J. L. Salak-Johnson, and P. A. Beck. 2023. Modified-live versus inactivated respiratory viral vaccines for revaccinating beef calves at weaning. App. Anim. Sci. 39. <https://doi.org/10.15232/aas.2022-02332>. |
| 15, 16 | Celestino, M. L., L. Fernandes, P. R. Menta, D. Paiva, T. L. Ribeiro, T. Silva, T. R. Bilby, R. C. Neves, M. A. Ballou, and V. S. Machado. 2020. The Effect of Metaphylactic Use of Tildipirosin for the Control of Respiratory Disease in Long-Distance Transported Dairy Calves. Front. Vet. Sci. 7. <https://doi.org/10.3389/fvets.2020.00632>. |
| 17, 28, 19, 20 | Crosby, W. B., B. B. Karisch, L. M. Hiott, J. D. Loy, W. B. Epperson, S. F. Capik, P. S. Morley, and A. R. Woolums. 2023. Tulathromycin metaphylaxis increases prevalence of multidrug resistant Mannheimia haemolytica while improving health in stocker calves. American Association of Bovine Practitioners Conference Proceedings. <https://doi.org/10.21423/aabppro20228647>. |
| 21, 22, 23 | Theurer, M. E., J. T. Fox, L. K. Bryant, J. S. Nickell, and J. P. Hutcheson. 2018. Treatment efficacy of tildipirosin or tulathromycin for first treatment of naturally occurring bovine respiratory disease in a commercial feedlot. Bov. Pract. (Stillwater). <https://doi.org/10.21423/bovine-vol52no2p154-159>. |
| 24, 25, 26 | Nickell, J., L. Bryant, K. F. Lechtenberg, and C. Cull. 2020. Evaluation of antimicrobial or non-antimicrobial treatments in commercial feedlot cattle with mild bovine respiratory disease based on a refined case-definition. Front Vet Sci. 7. <https://doi.org/10.3389/fvets.2020.571697>. |
| 27 | Pollreisz, J. P., C. Cull, K. Lechtenberg, T. Short, M. Blanding, J. Hinrichs, and H. D. Hughes. 2022. Comparison of two distinct arrival and treatment programs for bovine respiratory disease in high-risk feeder cattle entering a feedlot. Transl. Anim. Sci. 6. <https://doi.org/10.1093/tas/txac102>. |
| 28, 29, 30 | Coetzee, J. F., N. Cernicchiaro, P. K. Sidhu, and M. D. Kleinhenz. 2020. Association between antimicrobial drug class selection for treatment and retreatment of bovine respiratory disease and health, performance, and carcass quality outcomes in feedlot cattle. J. Anim. Sci. 98. <https://doi.org/10.1093/JAS/SKAA109>. |
| 31, 32 | Schumaher, T. F., R. F. Cooke, A. P. Brandão, K. M. Schubach, O. A. de Sousa, D. W. Bohnert, and R. S. Marques. 2019. Effects of vaccination timing against respiratory pathogens on performance, antibody response, and health in feedlot cattle. J. Anim. Sci. 97. <https://doi.org/10.1093/jas/sky466>. |
| 33, 34, 35 | Nickell, J. S., J. P. Hutcheson, D. G. Renter, and D. A. Amrine. 2021. Comparison of a traditional bovine respiratory disease control regimen with a targeted program based upon individualized risk predictions generated by the Whisper on Arrival technology. Transl. Anim. Sci. 5. <https://doi.org/10.1093/tas/txab081>. |
| 36, 37 | Dornbach, C. W., A. M. Beenken-Bobb, D. W. Shike, S. L. Hansen, and J. C. McCann. 2023. Effects of injectable vitamin E before or after transit on receiving phase growth performance, health, and blood parameters of beef steers. J. Anim. Sci. 101. <https://doi.org/10.1093/jas/skac333>. |
| 38 | Pickett, A. T., R. F. Cooke, I. S. de Souza, and S. J. Mackey. 2024. Administering the maternal bovine appeasing substance improves overall productivity and health in high-risk cattle during a 60-d feedlot receiving period. J. Anim. Sci. 102. <https://doi.org/10.1093/jas/skae221>. |
| 39, 40 | Silva, L. G. T., R. F. Cooke, K. M. Schubach, A. P. Brandão, R. S. Marques, T. F. Schumaher, P. Moriel, and D. W. Bohnert. 2018. Supplementing a yeast-derived product to enhance productive and health responses of beef steers. Animal. 12. <https://doi.org/10.1017/S1751731117003585>. |
| 41 | Rivera, J. D., J. T. Johnson, and M. D. Cravey. 2019. Effects of yeast and yeast cell wall on performance and health of newly received beef steers and heifers grazing bahiagrass pastures. App. Anim. Sci. 35. <https://doi.org/10.15232/aas.2018-01804>. |
| 42, 43 | Woolsoncroft, M. A., M. E. Youngers, L. J. McPhillips, C. G. Lockard, C. L. Haviland, E. S. DeSocio, W. R. Ryan, C. J. Richards, and B. K. Wilson. 2018. Effects of exercise and roughage source on the health and performance of receiving beef calves. Professional Animal Scientist. 34. <https://doi.org/10.15232/pas.2017-01673>. |
| 44 | Credille, B., R. D. Berghaus, E. Jane Miller, A. Credille, N. F. D. Schrag, and H. Naikare. 2024. Antimicrobial Metaphylaxis and its Impact on Health, Performance, Antimicrobial Resistance, and Contextual Antimicrobial Use in High-Risk Beef Stocker Calves. J. Anim. Sci. 102. <https://doi.org/10.1093/jas/skad417>. |
| 45, 46, 47 | Szasz, J. I., C. P. McMurphy, T. C. Bryant, J. Luque, C. Barcelo, G. Sepulveda, K. Shawn Blood, B. C. Bernhard, and H. D. Hughes. 2019. Influence of therapeutic use of feedgrade tetracyclines in combination with tulathromycin metaphylaxis on animal health and performance of Holstein steer calves. Transl. Anim. Sci. 3. <https://doi.org/10.1093/tas/txy135>. |
| 48 | Crosby, S., B. Credille, S. Giguère, and R. Berghaus. 2018. Comparative efficacy of enrofloxacin to that of tulathromycin for the control of bovine respiratory disease and prevalence of antimicrobial resistance in Mannheimia haemolytica in calves at high risk of developing bovine respiratory disease. J. Anim. Sci. 96. <https://doi.org/10.1093/jas/sky054>. |
| 49, 50 | Powledge, S. A., T. B. McAtee, A. R. Woolums, T. Robin Falkner, J. T. Groves, M. Thoresen, R. Valeris-Chacin, and J. T. Richeson. 2022. Clinical and microbiological effects in high-risk beef calves administered intranasal or parenteral modified-live virus vaccines. J. Anim. Sci. 100. <https://doi.org/10.1093/jas/skac249>. |
| 51 | Rivera, J. D., J. T. Johnson, and G. K. Blue. 2018. Effects of oral tilmicosin on health and performance in newly received beef heifers. Professional Animal Scientist. 34. <https://doi.org/10.15232/pas.2017-01639>. |
| 52 | Nelson, W. T., M. E. Branine, T. C. Bryant, S. L. Archibeque, and J. J. Wagner. 2022. Evaluation of an experimental bio-nutrient feedlot premix on health, growth performance, carcass merit, and prevalence and severity of liver abscesses in feedlot cattle managed under a natural feeding protocol. App. Anim. Sci. 38. <https://doi.org/10.15232/aas.2021-02244>. |
| 53, 54 | Peterson, S. J., B. L. Nuttelman, D. B. Burken, M. K. Luebbe, G. E. Erickson, and J. C. MacDonald. 2015. Use of a pelleted corn-residue complete feed in calf receiving diets. Professional Animal Scientist. 31. <https://doi.org/10.15232/pas.2014-01363>. |
| 55 | Cheek, R. A., E. B. Kegley, J. R. Russell, J. L. Reynolds, K. A. Midkiff, D. Galloway, and J. G. Powell. 2024. Supplemental trace minerals as complexed or inorganic sources for beef cattle during the receiving period. J. Anim. Sci. 102. <https://doi.org/10.1093/jas/skae056>. |
| 56, 57, 58 | Deters, E. L., and S. L. Hansen. 2019. Vitamin E supplementation strategies during feedlot receiving: Effects on beef steer performance, antibody response to vaccination, and antioxidant defense. J. Anim. Sci. 97. <https://doi.org/10.1093/jas/skz289>. |
| 59, 60, 61 | Richeson, J. T., P. A. Beck, H. D. Hughes, D. S. Hubbell, M. S. Gadberry, E. B. Kegley, J. G. Powell, and F. L. Prouty. 2015. Effect of growth implant regimen on health, performance, and immunity of high-risk, newly received stocker cattle. J. Anim. Sci. 93. <https://doi.org/10.2527/jas.2014-8835>. |
| 62, 63, | Ryan, A. W., E. B. Kegley, J. Hawley, J. G. Powell, J. A. Hornsby, J. L. Reynolds, and S. B. Laudert. 2015. Supplemental trace minerals (zinc, copper, and manganese) as sulfates, organic amino acid complexes, or hydroxy trace-mineral sources for shipping-stressed calves. Professional Animal Scientist. 31. <https://doi.org/10.15232/pas.2014-01383>. |
| 64, 65 | Palmer, E. A., E. B. Kegley, J. J. Ball, P. A. Beck, J. A. Hornsby, J. L. Reynolds, B. P. Shoulders, A. R. Boyer, and J. G. Powell. 2019. Influence of commercial yeast products in diets for beef cattle new to the feedlot environment. App. Anim. Sci. 35. <https://doi.org/10.15232/aas.2019-01847>. |
| 66, 67 | Word, A. B., T. A. Wickersham, L. A. Trubenbach, G. B. Mays, and J. E. Sawyer. 2020. Effects of metaphylaxis on production responses and total antimicrobial use in high-risk beef calves. App. Anim. Sci. 36. <https://doi.org/10.15232/aas.2019-01914>. |
| 68 | White, B. J., D. E. Amrine, and D. R. Goehl. 2015. Determination of value of bovine respiratory disease control using a remote early disease identification system compared with conventional methods of metaphylaxis and visual observations. J. Anim. Sci. 93. <https://doi.org/10.2527/jas.2015-9079>. |
| 69 | Roberts, S. L., N. D. May, C. L. Brauer, W. W. Gentry, C. P. Weiss, J. S. Jennings, and J. T. Richeson. 2016. Effect of injectable trace mineral administration on health, performance, and vaccine response of newly received feedlot cattle. Professional Animal Scientist. 32. <https://doi.org/10.15232/pas.2016-01543>. |
| 70, 71, 72 | Munoz, V. I., K. L. Samuelson, D. J. Tomczak, H. A. Seiver, T. M. Smock, and J. T. Richeson. 2020. Comparative efficacy of metaphylaxis with tulathromycin and pentavalent modified-live virus vaccination in high-risk, newly received feedlot cattle. App. Anim. Sci. 36. <https://doi.org/10.15232/aas.2020-02054>. |
| 73, 74 | Homolka, M. N., W. N. Smith, T. C. Husz, D. M. Paulus-Compart, and J. S. Jennings. 2023. Evaluating performance of beef cattle consuming a supplemental probiotic-prebiotic during the first 21 or 42 days after feedlot arrival. Livest Sci. 270. <https://doi.org/10.1016/j.livsci.2022.105096>. |
| 75, 76 | Galyen, W. L., P. A. Beck, T. W. Hess, D. S. Hubbell, M. S. Gadberry, E. B. Kegley, M. Cravey, and J. G. Powell. 2020. Effects of bambermycin with amprolium or monensin on beef cattle growth performance, coccidia infection, and bovine respiratory disease morbidity during receiving. App. Anim. Sci. 36. <https://doi.org/10.15232/aas.2019-01971>. |
| 77 | Ball, J. J., E. B. Kegley, J. Sarchet, and J. G. Powell. 2019. Comparison of treatment protocols for bovine respiratory disease in high-risk, newly received beef calves. Applied Animal Science. 35. <https://doi.org/10.15232/aas.2018-01836>. |
| 78 | Horton, L. M., B. E. Depenbusch, D, M. Dewsbury, T. B. McAtee, N. B. Betts, and D. G. Renter. 2023. Comprehensive outcomes affected by antimicrobial metaphylaxis of feedlot calves at medium-risk for bovine respiratory disease from a randomized controlled trial. Vet. Sci. 10:67. <https://doi.org/10.3390/vetsci10020067> |
| 79, 80, 81 | Coppin, C. M., T. M. Smock, C. L. Helmuth, J. L. Manahan, N. S. Long, A. A. Hoffman, J. A. Carroll, P. R. Broadway, N. C. Burdick Sanchez, J. E. Wells, S. C. Fernando, and K. E. Hales. 2022. The effects of administering different metaphylactic antimicrobials on growth performance and health outcomes of high-risk, newly received feedlot steers. Transl. Anim. Sci. 6. <https://doi.org/10.1093/tas/txac140>. |
| 82, 83 | Lippolis, K. D., R. F. Cooke, T. Schumaher, A. P. Brandão, L. G. T. Silva, K. M. Schubach, R. S. Marques, and D. W. Bohnert. 2017. Physiologic, health, and performance responses of beef steers supplemented with an immunomodulatory feed ingredient during feedlot receiving. J. Anim. Sci. 95. <https://doi.org/10.2527/jas2017.1837>. |
| 84, 85 | Gouvêa, V. N., M. O. Oliveira, H. J. M. Giacomelli, E. A. Colombo, F. Batistel, F. A. P. Santos, G. C. Duff, R. S. Marques, and R. F. Cooke. 2023. Roughage level and supplemental fat for newly received finishing calves: effects on growth performance, health, and physiological responses. J. Anim Sci. 101. <https://doi.org/10.1093/jas/skac322>. |
| 86, 87 | Smock, T. M., P. R. Broadway, N. C. Burdick Sanchez, J. A. Carroll, A. A. Hoffman, N. S. Long, J. L. Manahan, Z. S. McDaniel, M. E. Theurer, J. E. Wells, and K. E. Hales. 2023. Infrared thermography or rectal temperature as qualification for targeted metaphylaxis in newly received beef steers and the effects on growth performance, complete blood count, and serum haptoglobin during a 42-day feedlot receiving period*. App. Anim. Sci. 39. <https://doi.org/10.15232/aas.2022-02370>. |
| 88, 89 | Wiegand, J. B., R. F. Cooke, A. P. Brandão, K. M. Schubach, E. A. Colombo, C. L. Daigle, G. C. Duff, and V. N. Gouvêa. 2020. Impacts of commingling cattle from different sources on their physiological, health, and performance responses during feedlot receiving. Transl. Anim. Sci. 4. <https://doi.org/10.1093/tas/txaa204>. |
| 90 | Colombo, E. A., R. F. Cooke, A. P. Brandão, J. B. Wiegand, K. M. Schubach, G. C. Duff, V. N. Gouvêa, and B. I. Cappellozza. 2020. Administering an appeasing substance to optimize performance and health responses in feedlot receiving cattle. J. Anim. Sci. 98. <https://doi.org/10.1093/JAS/SKAA339>. |
| 91, 92 | Colombo, E. A., R. F. Cooke, A. P. Brandão, J. B. Wiegand, K. M. Schubach, C. A. Sowers, G. C. Duff, E. Block, and V. N. Gouvêa. 2021. Performance, health, and physiological responses of newly received feedlot cattle supplemented with pre- and probiotic ingredients. Animal. 15. <https://doi.org/10.1016/j.animal.2021.100214>. |
| 93 | Tomczak, D. J., K. L. Samuelson, J. S. Jennings, and J. T. Richeson. 2019. Oral hydration therapy with water affects health and performance of high-risk, newly received feedlot cattle. App. Anim. Sci. 35. <https://doi.org/10.15232/aas.2018-01796>. |
| 94 | Idowu, M. D., G. Taiwo, A. Pech Cervantes, S. A. Bowdridge, and I. M. Ogunade. 2022. Effects of a multicomponent microbial feed additive containing prebiotics and probiotics on health, immune status, metabolism, and performance of newly weaned beef steers during a 35-d receiving period. Transl. Anim. Sci. 6. <https://doi.org/10.1093/tas/txac053>. |
| 95, 96 | Cordeiro, M. W. S., B. I. Cappellozza, N. N. de Melo, and T. F. Bernardes. 2024. Effects of a Bacillus-based direct-fed microbial on performance, blood parameters, fecal characteristics, rumen morphometrics, and intestinal gene expression in finishing beef bulls. J. Anim. Sci. 102. <https://doi.org/10.1093/jas/skae259>. |
| 97, 98 | Duff, G. C., K. J. Malcolm-Callis, D. A. Walker, M. W. Wiseman, M. L. Galyean, and L. J. Perino. 2019. Effects of Intranasal Versus Intramuscular Modified Live Vaccines and Vaccine Timing on Health and Performance by Newly Received Beef Cattle. Bov. Pract. (Stillwater). <https://doi.org/10.21423/bovine-vol34no1p66-71>. |
| 99, 100 | Van Engen, N. K., T. J. Engelken, C. G. Lockard, J. Lakritz, N. Cernicchiaro, B. K. Wilson, C. R. Krehbiel, and J. F. Coetzee. 2019. The effects of pretransportation or arrival meloxicam administration to calves entering the feedlot on morbidity, biomarkers, performance, and carcass characteristics. Transl. Anim. Sci. 3. <https://doi.org/10.1093/tas/txz070>. |
| 101, 102, 103 | Smock, T. M., K. L. Samuelson, J. E. Hergenreder, P. Whitney Rounds, and J. T. Richeson. 2020. Effects of Bacillus subtilis PB6 and/or chromium propionate supplementation on clinical health, growth performance, and carcass traits of high-risk cattle during the feedlot receiving and finishing periods. Transl. Anim. Sci. 4. <https://doi.org/10.1093/tas/txaa163>. |
